# Sequence analysis and spatiotemporal developmental distribution of the Cat-1-type transporter *slc7a1a* in zebrafish (*Danio rerio*)

**DOI:** 10.1007/s10695-020-00873-x

**Published:** 2020-09-27

**Authors:** Ståle Ellingsen, Shailesh Narawane, Anders Fjose, Tiziano Verri, Ivar Rønnestad

**Affiliations:** 1 Department of Molecular Biology, University of Bergen, Postbox 7803, NO-5020 Bergen, Norway; 2 Present Address: Department of Biological Sciences, University of Bergen, Postbox 7803, NO-5020 Bergen, Norway; 3 Department of Biological and Environmental Sciences and Technologies, University of Salento, via Prov.le Lecce-Monteroni, I-73100 Lecce, Italy

**Keywords:** Cationic amino acid transport(ers), *slc7a1a*, *slc7a1b*, Spatiotemporal expression, Zebrafish

## Abstract

Cationic amino acid transporter 1 (Cat-1 *alias* Slc7a1) is a Na^+^-independent carrier system involved in transport and absorption of the cationic amino acids lysine, arginine, histidine, and ornithine and has also been shown to be indispensable in a large variety of biological processes. Starting from isolated full-length zebrafish (*Danio rerio*) cDNA for *slc7a1a*, we performed comparative and phylogenetic sequence analysis, investigated the conservation of the gene during vertebrate evolution, and defined tissue expression during zebrafish development. Whole mount in situ hybridization first detected *slc7a1a* transcripts in somites, eyes, and brain at 14 h post-fertilization (hpf) with additional expression in the distal nephron at 24 hpf and in branchial arches at 3 days post-fertilization (dpf), with significant increase by 5 dpf. Taken together, the expression analysis of the zebrafish Cat-1 system gene *slc7a1a* suggests a functional role(s) during the early development of the central nervous system, muscle, gills, and kidney.

**Electronic supplementary material:**

The online version of this article (10.1007/s10695-020-00873-x) contains supplementary material, which is available to authorized users.

**Figure:**
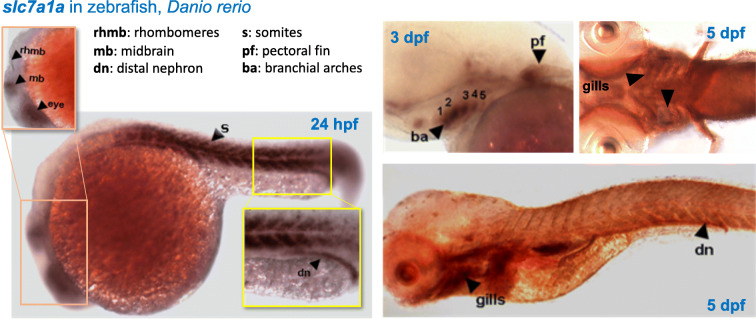
Graphical abstract

## Introduction

The cationic amino acid transporter 1 (Cat-1), a member of the solute carrier (SLC) 7 family of proteins, also referred to as solute carrier family 7 member 1 (Slc7a1), is a Na^+^-independent transporter of cationic amino acids. In mammals, the Cat protein group consists of Cat-1, Cat-2a, Cat-2b, and Cat-3, all exhibiting a nearly identical substrate specificity for cationic l-amino acids (Deves et al. 1998; Closs et al. 2004; Closs et al. 2006). Cat-4 is acknowledged to have no affinity for cationic, neutral, or acidic amino acids and is referred to as an orphan transporter, similarly to the Cat protein referred to as Slc7a14 (Closs et al. 2006). So far, very little information is available on the molecular and functional characterizations of these transporters in teleost fish (Gu et al. 2014).

Cat proteins are predicted to have 14 transmembrane (TM) regions, with intracellular N- and C-termini (Albritton et al. 1989). CAT proteins show a variable number of amino acids, also due to the occurrence of alternative splicing events, see, e.g., human SLC7A1 (629 amino acids), SLC7A2A (657 amino acids), SLC7A2B (658 amino acids), SLC7A3 (619 amino acids), SLC7A4 (635 amino acids), and SLC7A14 (711 amino acids) (Closs et al. 2006).

Special features of all Cat proteins include a glutamic acid residue (E^107^) located at the intracellular face of the third TM, which is recognized to be necessary for the transport activity of mouse Slc7a1 (Wang et al. 1994). Also, the third extracellular loop of mouse Slc7a1, which acts as an ecotropic murine leukemia virus binding site (Albritton et al. 1993), contains two asparagine residues (N^223^ and N^229^) that are glycosylated in mouse Slc7a1 (Kim and Cunningham 1993). These N-linked glycosylation sites are conserved in mouse and human proteins (Wang et al. 1996).

Cats mediate bidirectional cationic amino acid transport and support important physiological functions such as protein synthesis and inter-organ amino acid flow (Hatzoglou et al. 2004). Through arginine homeostasis, Cats are also involved in nitric oxide (NO) synthesis, polyamine biosynthesis, and collagen synthesis (Hatzoglou et al. 2004; Closs et al. 2006; North et al. 2009). In addition, in mammals, the *SLC7A1*/*Slc7a1* gene is vital for cell survival during stress as it permits cells to recommence growth as soon as amino acids become available (Hatzoglou et al. 2004; Closs et al. 2006).

Extensive studies in mammalian models have reported that *SLC7A1* is associated with endothelial NOS (eNOS) and caveolin in pulmonary artery endothelial cells (McDonald et al. 1997) and that it is localized to the basolateral membrane in polarized Madin-Darby canine kidney (MDCK) and human embryonic kidney 293 (HEK293) cells (Cariappa et al. 2002; Kizhatil and Albritton 2002). Human keratinocytes constitutively express *SLC7A1* and *SLC7A2* that mediate arginine influx essential for both inducible NOS (iNOS) and arginase enzyme activities, which eventually modulate proliferation and differentiation of epidermal skin cells (Schnorr et al. 2003). In addition, *SLC7A1* is expressed in the brain microvascular endothelial cells at the human blood brain barrier (Umeki et al. 2002), and it localizes in retinal capillary endothelial cells where it facilitates arginine transport at the inner blood retinal barrier (Tomi et al. 2009). Functionally, SLC7A1-mediated arginine import is essential for both differentiation and proliferation of erythrocytes (Shima et al. 2006). *Slc7a1* loss-of-function in mice leads to anemia (Perkins et al. 1997) and 25% size reduction compared with wild-type littermates and perinatal death (Nicholson et al. 1998). Overall, *SLC7A1/Slc7a1* distributes differentially among different cells, with varying affinities for basic amino acids arginine, lysine, and ornithine (MacLeod 1996; Closs et al. 2004). Little information is available on changes in *SLC7A1*/*Slc7a1* expression during embryonic development. Notably, amino acid transport promotes preimplantation mouse embryo development at different stages, e.g., nonessential amino acid transport improves development mainly during cleavage, while essential amino acid transport supports development after the eight-cell stage (Van Winkle 2001).

In this context and with special regard to the elucidation of the role of basic amino acid transport processes in teleost systems and compartments, where at least to our knowledge no information is available to date, we isolated the full-length *slc7a1a* in zebrafish (*Danio rerio*) and focused on its spatial expression during the early stages of development. Our findings revealed conservation of functionally important amino acids in the zebrafish *slc7a1a* sequence along with the conservation of synteny, which suggests a common biochemical scheme in basic amino acid transport processes across teleost fish membranes, and from teleost fish to mammals. Also, in situ hybridization revealed embryonic and larval stage specific *slc7a1a* expression in eyes, somites, distal nephrons, and branchial arches. Taken together, these results suggest that *slc7a1a* has embryonic stage and organ specific expression and is possibly important for normal embryonic development and function.

## Materials and methods

### Zebrafish maintenance

Zebrafish were maintained and bred at HIB, University of Bergen, as described elsewhere (Stuart et al. 1988). Zebrafish embryos were obtained from natural mating, and pigmentation was prevented by adding 0.003% phenylthiourea (PTU) to E3 medium (5 mM NaCl, 0.17 mM KCl, 0.33 mM CaCl_2_, 0.33 mM MgSO_4_).

### Sequence analysis

The nucleotide and protein sequences described in this study were obtained from Ensembl (http://www.ensembl.org/). Predicted transcripts used for *slc7a1a* isolation refer to Ensembl Transcript ID: ENSDART00000008248 (Ensembl Gene ID: ENSDARG00000016439). The cloned zebrafish *slc7a1a* was sequenced and the translated sequence used for sequence comparisons.

The SLC7A1/Slc7a1-type amino acid sequences used for sequence comparison are in Table S1 (see also Appendix I in Supplementary Material). Multiple protein sequence alignments were performed using Clustal Omega (https://www.ebi.ac.uk/Tools/msa/clustalo/). The neighbor-joining (NJ) method-based phylogenetic tree was built using MEGA X (http://www.megasoftware.net).

Putative transmembrane domains were predicted using TMHMM 2.0 (http://www.cbs.dtu.dk/services/TMHMM/), which is part of the Simple Modular Architecture Research Tool (SMART) (http://smart.embl-heidelberg.de/). Potential *N*-glycosylation and protein kinase C recognition sequences were identified using the PROSITE 19.7 computational tools (http://www.expasy.org/prosite/).

Conservation of the zebrafish *slc7a1a* gene with respect to other vertebrate orthologous/paralogous genes (synteny) was evaluated by gene database consulting at the National Center for Biotechnology Information (NCBI) (https://www.ncbi.nlm.nih.gov/gene).

### Sequence isolation

**Fig. 1:**
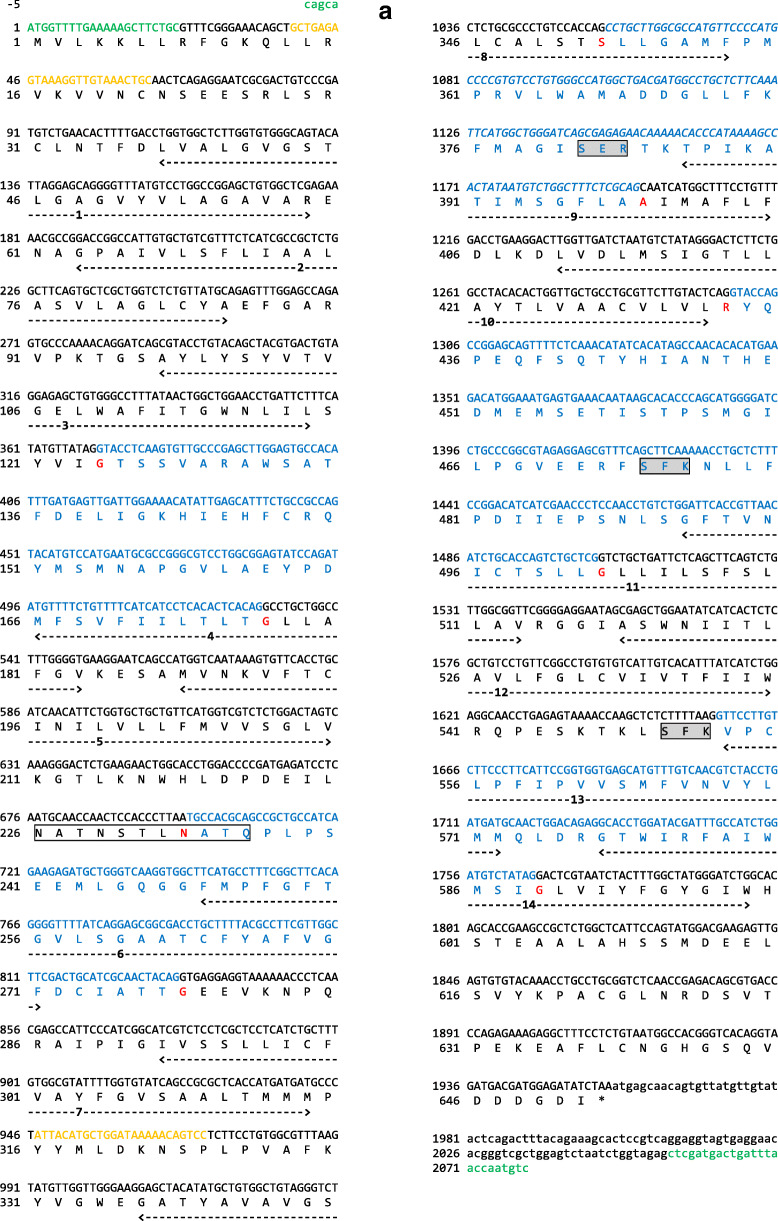

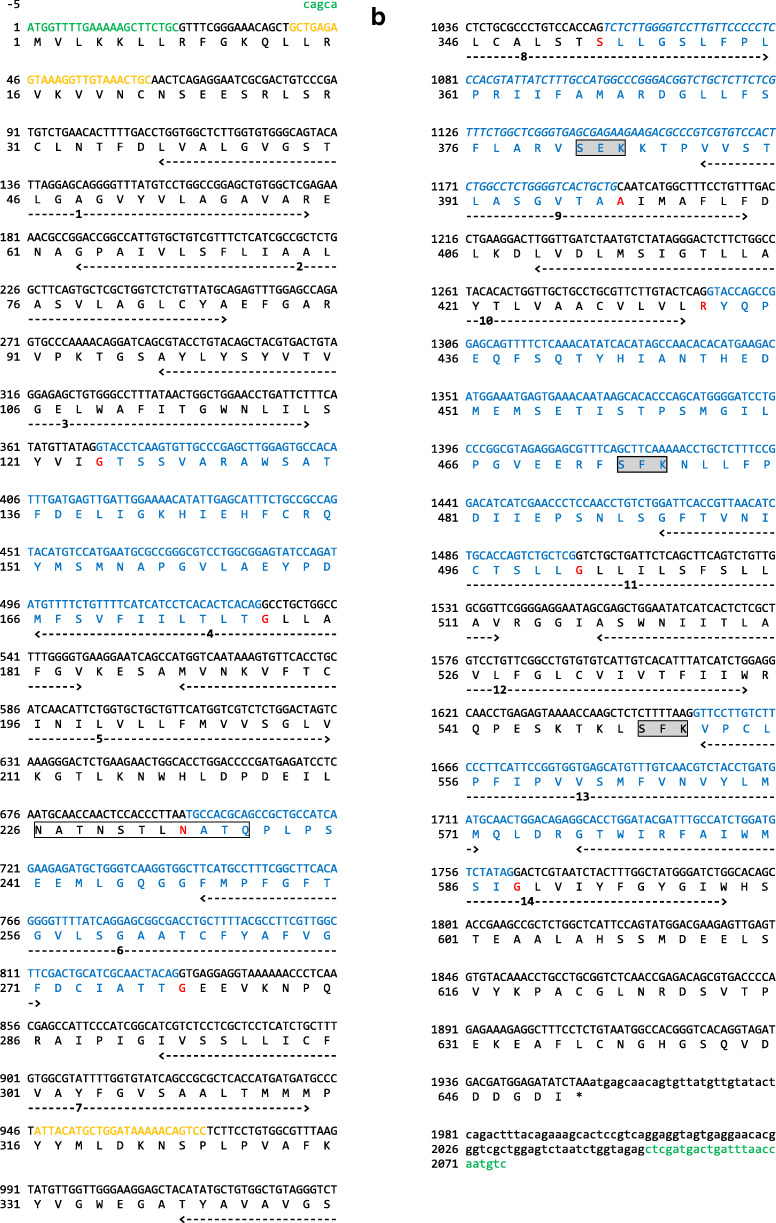

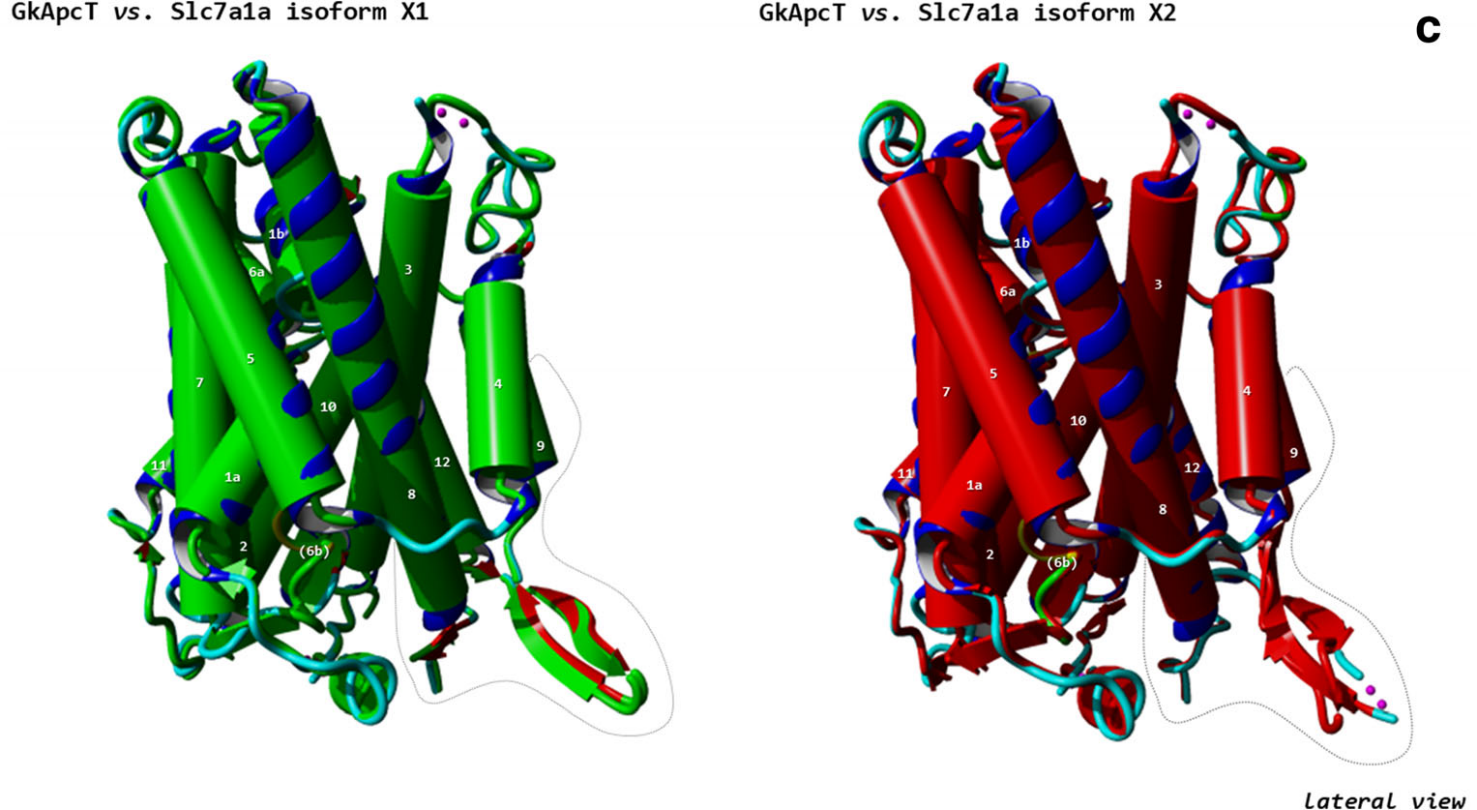
Nucleotide and predicted amino acid sequence of zebrafish *slc7a1a*. The figure was generated using ORFfinder (https://www.ncbi.nlm.nih.gov/orffinder/). Numbers on the left refer to the nucleotide (upper row) and amino acid (lower row) positions. Nucleotides are numbered, starting from the first ATG initiation codon. Asterisk indicates the stop codon. The specific primers used for full-length cDNA cloning and whole mount in situ hybridization probe generation (see also Table S2) are indicated in green and orange, respectively. In the amino acid sequence, putative transmembrane domains, obtained using the TMHMM v. 2.0 program as implemented in SMART, are indicated by arrows and named 1 to 14. Potential extracellular N-glycosylation sites (white boxes) and potential protein kinase C phosphorylation sites at the cytoplasmic surface (dark gray boxes) were obtained using the ScanProsite tool. Coding exons are drawn in black and blue. The alternatively spliced forms of coding exon 6 (exon 6a and exon 6b) are drawn in italics. **a** Slc7a1a isoform X1. **b** Slc7a1a isoform X2. **c** Three-dimensional appearance (lateral view) of zebrafish Slc7a1a proteins (isoform X1 and isoform X2). Homology modeling by Phyre^2^ (intensive mode) was used to predict the structures. The structure of a proton-coupled amino acid transporter with the leucine transporter (LeuT) fold from *Geobacillus kaustophilus* (GkApcT) (Protein Data Bank Acc. No. 5OQT) was the template. For both isoforms, confidence (i.e., the probability that the match between the query sequence and the template is a true homology and thus that the template is correct) reached the value of 100% (the highest accuracy) and the residues in the model covered 71% of the corresponding experimental structure. After pairwise structure alignment (TM-align), the superimposed structures (i.e., GkApcT vs. Slc7a1a isoform X1 or Slc7a1a isoform X2) were drawn (YASARA View). Putative transmembrane domains are named 1 to 12 (please note that transmembrane domains 1 and 6 are indicated as 1a and 1b and 6a and 6b, respectively). For each protein, the region of amino acid sequence change due to the alternative splicing spans from approximately the second half of transmembrane domain 8 to the first half of transmembrane domain 9 through a cytoplasmic beta hairpin loop (delimited by a dashed line)

A *slc7a1a* cDNA was cloned from total RNA retro-transcription followed by PCR amplification (as described previously; Rønnestad et al. 2010) using a specific primer pair encompassing the open reading frame (ORF) (for details, see Fig. 1 a and b; for primers, see also Table S2). The in situ probes for *slc7a1a* were prepared by PCR amplification of a *slc7a1a* cDNA fragment (for details, see Fig. 1 a and b; for primers, see also Table S2). The PCR products were cloned into a pCRII-TOPO vector (Invitrogen, Germany). All sequences were verified by sequencing.

### Protein modeling

Phyre^2^ (Kelley et al. 2015) in intensive mode (http://www.sbg.bio.ic.ac.uk/phyre2/html/page.cgi?id=index) was used to predict the structures of the zebrafish Slc7a1a proteins (isoforms X1 and X2) from their sequences (for details, see Fig. 1c; see also Appendix I in Supplementary Material). Analogously, Phyre^2^ was used to predict the structures of all the SLC7-type proteins used for comparison (for details, see Table 1 and Fig. 4c; see also Appendix I in Supplementary Material). Whenever required, pairwise structure alignments were generated by TM-align (Zhang and Skolnick 2005) at https://zhanglab.ccmb.med.umich.edu/TM-align/. YASARA View (Krieger and Vriend 2014) was used to visualize all the various three-dimensional structures.

**Table 1:** The solute carrier 7 family members of the so-called cationic amino acid transporters group in human (*Homo sapiens*) compared with zebrafish (*Danio rerio*)

From: http://www.bioparadigms.org						From: http://www.guidetopharmacology.org	From: http://www.ncbi.nlm.nih.gov/gene	From: https://www.ncbi.nlm.nih.gov/unigene/	From: http://zfin.org		
SLC name	Protein name	Aliases	Transport type	Substrates	Tissue and cellular expression	Substrates	SLC name	EST profile	Tissue and cellular expression	Stage range	References
SLC7A1	CAT-1	ATRC1, system y^+^	F (non-obligatory E)	Cationic l-amino acids	Ubiquitous except for liver, lacrimal gland	l-arginine, l-lysine, l-ornithine, l-histidine	*slc7a1a* (*Chr10*)	Developmental stage | adult Adult | heart > reproductive system	This study	This study	This study
							*slc7a1b* (alias *zgc:63694*) (*Chr 15*)	Developmental stage | gastrula > juvenile > hatching > adult Adult | reproductive system > muscle	Whole organism	1-cell to Pec-fin	Thisse and Thisse (2004)
SLC7A2	CAT-2 (A or B)	ATRC2, system y^+^	F	Cationic l-amino acids	CAT-2A: liver, skeletal muscle, pancreas, CAT-2B: inducible in many cell types	l-arginine, l-lysine, l-ornithine, l-histidine	*slc7a2* (*Chr 14*)	-	Unspecified (possibly pancreas, presumptive skull, cells at the border of cornea)	20–25 somites to day 5	Thisse and Thisse (2004)
							*zgc:175280* (*Chr 5*)	Developmental stage | adult Adult | intestine > kidney	-	-	-
SLC7A3	CAT-3	ATRC3, system y^+^	F	Cationic l-amino acids	Thymus, ovary, testis, brain (neurons)	l-arginine, l-lysine, l-ornithine	*slc7a3a* (*Chr 21*)	Developmental stage | adult > pharyngula > hatching Adult | kidney > eye > fin > reproductive system > muscle > brain	Head, whole organism	1-cell to protruding-mouth	Thisse and Thisse (2004) Gu et al. (2014)
							*slc7a3b* (*Chr 14*)	Developmental stage | adult Adult | muscle > brain	-	-	-
SLC7A4	CAT-4	-	O	-	Brain, testis, placenta	-	*slc7a4* (*Chr 8*)	Developmental stage | adult Adult | gills > reproductive system > eye	-	-	-
SLC7A14			O		Highly expressed in CNS	-	*slc7a14a* (*Chr 2*)	**-**	-	-	Jin et al. (2014)
							*slc7a14b* (*Chr 24*)	**-**	-	-	-

Abbreviations for transport type: *E* exchanger, *F* facilitated transporter, *O* orphan transporter

### RNA probe preparation and DIG-labeling

Plasmid DNA was linearized with appropriate restriction endonucleases for 5 h at 37 °C, purified using QIAquick Nucleotide Removal Kit (Qiagen, Hilden, Germany), and the degree of linearization was examined on a 1% agarose gel. In vitro transcription to produce digoxigenin (DIG)-labeled RNA probe was carried out combining linearized plasmid, 1 μg DIG labeling mix (Roche, Mannheim, Germany), 2 μl transcription buffer, 2 μl RNase inhibitor (Roche, Mannheim, Germany), 1 μl T7/Sp6 RNA polymerase (Roche), and 2 μl RNase-free ddH_2_O to a final volume of 20 μl. The mix was incubated at 37 °C for 2 h. This was followed by DNase I treatment for 15 min at 37 °C. Labeled RNA was purified using the RNeasy Mini Kit (Qiagen, Hilden, Germany); probe length was verified by agarose gel and then dissolved in 150 μl hybridization buffer and stored at − 20 °C until use.

### In situ hybridization

Zebrafish embryos, collected at 24 hpf, 3 dpf, and 5 dpf, were dechorionated, anesthetized with tricaine, washed with E3, and fixed overnight in 4% paraformaldehyde (PFA) at 4 °C. Twelve-fourteen hours post-fertilization embryos were fixed in 4% PFA before dechorionation. Pigmentation was prevented using 0.003% PTU in E3 medium. Fixed embryos were placed in 100% methanol at − 20 °C until use.

Whole mount in situ hybridization was carried out as described previously (Seo et al. 1998). Briefly, the DIG-labeled sense and antisense zebrafish *slc7a1* RNA probes were used, the former as negative control.

### Imaging

In situ hybridization images were captured with Leica M420™ and Nikon EPI-FL3™ microscope equipped with micropublisher 5.0 RTV camera (QImaging). Figures were generated using Adobe CS2 Photoshop™ and Illustrator™.

### Ethical treatment of animals

Zebrafish were maintained and experiments conducted in compliance with the Norwegian Animal Welfare Act guidelines. No ethical permission was needed. According to the EU Directive 2010/63/EU on the protection of animals used for scientific purposes, implemented in Norwegian legislation as of December 12, 2014, early life stages of zebrafish are not protected as animals until the stage of being capable of independent feeding, i.e., 5 days post fertilization (dpf).

## Results

### Cat-1 conservation among vertebrates

Detailed sequence analysis and interspecies comparison among vertebrates were performed to ascertain the identity of the cloned zebrafish Cat-1-type transporter gene and its organization. As assessed by GenBank database consulting (December 2019), two *slc7a1*-type genes are present in the zebrafish genome (GRCz11 Assembly) (Table 1), namely *slc7a1a* (also known as *slc7a1*; the gene analyzed in this study) and *slc7a1b* (also known as *zgc:63694*). Both genes produce “predicted” alternative splicing mRNA forms. In the case of *slc7a1a*, there are four predicted transcript variants in GenBank, indicated as X1 (Acc. N. XM_678531.7), X2 (Acc. N. XM_021479384.1), X3 (Acc. N. XM_005155277.4), and X4 (Acc. N. XR_002459600.1), and three predicted transcript variants in Ensembl, indicated as -201 (Transcript ID ENSDART00000008248.9), -202 (Transcript ID ENSDART00000146370.3), and -203 (Transcript ID ENSDART00000183510.1). These converge into two predicted alternative amino acid sequences in GenBank, indicated as isoforms X1 (e.g., Acc. N. XP_683623.4) and X2 (e.g., XP_005155334.1), and into three predicted alternative amino acid sequences in Ensembl, indicated as -201 (Protein ID ENSDARP00000027285.8), -202 (Protein ID ENSDARP00000121637.1), and -203 (Protein ID ENSDARP00000152628.1) (Fig. 1a, b; Table S1). The cloned full-length cDNA did code for the *slc7a1a* mRNA indicated as transcript variant X3 and coding for the Slc7a1a protein indicated as isoform X2 (Fig. 1b). In particular, the cloned zebrafish *slc7a1a* cDNA was 2081 nucleotides long, with an ORF of 1950 nucleotides coding a putative protein of 650 amino acids (Fig. 1b). Hydropathy analysis predicted 14 potential transmembrane domains with a large intracellular loop between transmembrane domains 10 and 11 (Fig. 1a and b). Such hydropathy findings were largely confirmed by a parallel analysis of the three-dimensional structures of zebrafish Slc7A1a (isoform X1 and isoform X2) proteins generated by homology modeling on a proton-coupled amino acid transporter with the leucine transporter (LeuT) fold from *Geobacillus kaustophilus* (GkApcT) (Protein Data Bank Acc. No. 5OQT). However, only 12 transmembrane domains occurred in these models, with transmembrane domains 11 and 12 of GkApcT corresponding to transmembrane domains 13 and 14 of the zebrafish Slc7a1a proteins (see hydropathy profile in Fig. 1a and b), due to the lack of superimposition in those parts of the zebrafish Slc7a1a proteins corresponding to transmembrane domains 11 and 12. Moreover, comparing zebrafish Slc7a1a isoform X2 to zebrafish Slc7a1a isoform X1 revealed differences in the protein sequence (amino acid positions X1:352–399 and X2:352–398) and length (X1: 651 vs. X2: 650 amino acids), due to an alternative splicing event involving coding exons 6a and 6b (Fig. 1a, b, and c; Fig. S1; for comparison, see also Fig. 4a, b, and c). Such differences were observed in other teleost fish Slc7a1a proteins analyzed in this study (Fig. S1).

**Fig. 2:**
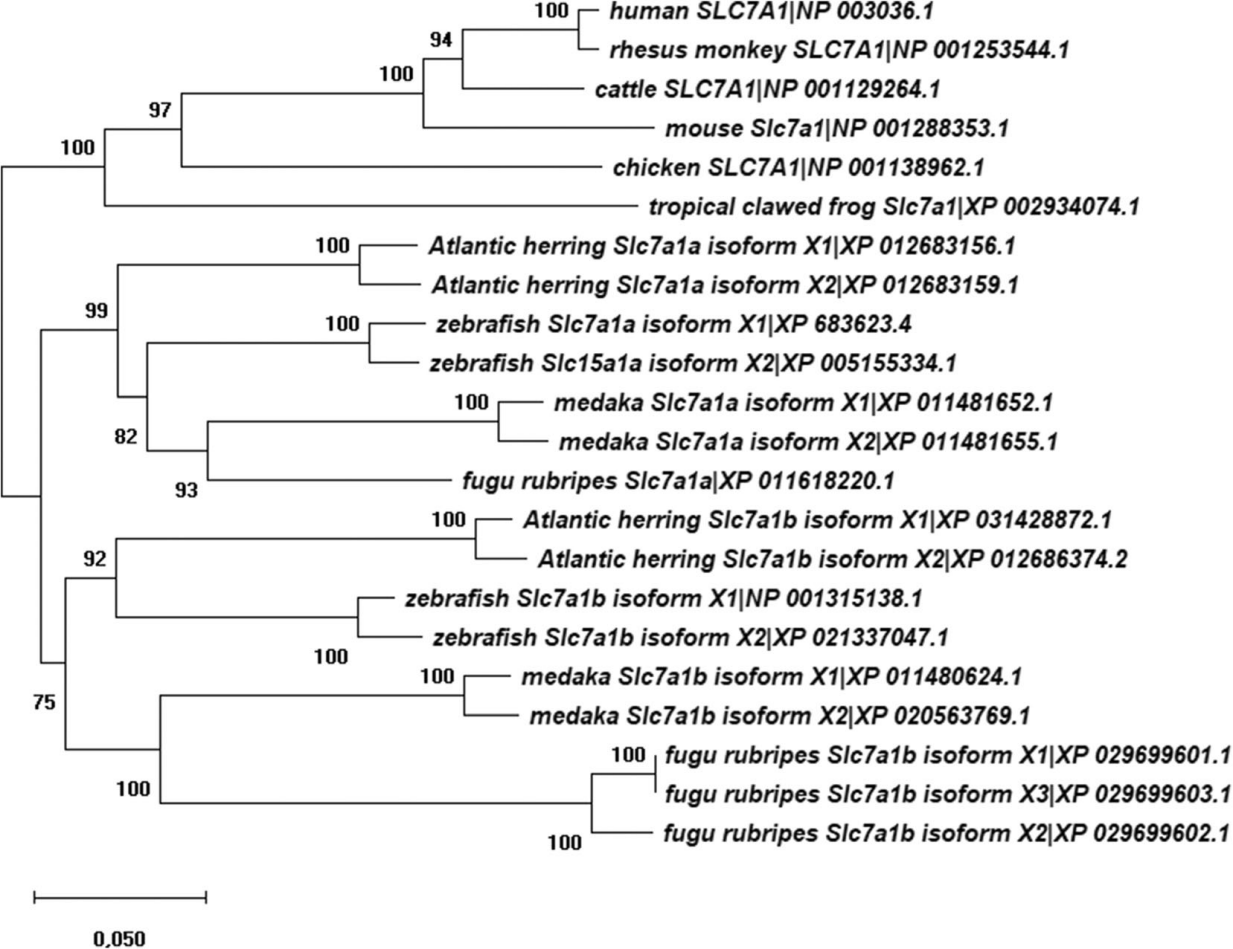
Slc7a1a evolutionary relationships of taxa. The evolutionary history was inferred using the neighbor-joining method (Saitou and Nei 1987). The optimal tree with the sum of branch length = 157,066,916 is shown. The percentage of replicate trees in which the associated taxa clustered together in the bootstrap test (1000 replicates) is shown next to the branches (Felsenstein 1985). The tree is drawn to scale, with branch lengths in the same units as those of the evolutionary distances used to infer the phylogenetic tree. The evolutionary distances were computed using the Poisson correction method (Zuckerkandl and Pauling 1965) and are in the units of the number of amino acid substitutions per site. The analysis involved 22 amino acid sequences. All positions containing gaps and missing data were eliminated. There were a total of 579 positions in the final dataset. Evolutionary analyses were conducted in MEGA X (Kumar et al. 2018)

Predicted Slc7a1-type amino acid sequences from zebrafish, Atlantic herring, medaka, fugu rubripes, tropical clawed frog, chicken, cattle, mouse, rhesus monkey, and human were aligned using Clustal Omega, and phylogenetic tree analysis was performed (Fig. 2). The phylogenetic tree construction revealed branching of Slc7a1-type proteins from teleost fish species in one cluster while the mammalian species branched in a cluster together with chicken and tropical clawed frog. Coherently, teleost fish Slc7a1a and Slc7a1b proteins clustered in two separate branches, suggesting gene duplication occurrence in this lower vertebrate group (Fig. 2). Alignment of the zebrafish Slc7a1a sequences (Slc7a1a isoform X1: 97.0; Slc7a1a isoform X2: 100%) revealed a slightly higher percentage of identity with Slc7a1a sequences from the other teleost fish (Atlantic herring 79.4–80.8%; medaka 78.5–79.8%; fugu rubripes 80.1%) than with any sequences from amphibians (tropical clawed frog Slc7a1 68.3%), birds (chicken SLC7A1 72.1%), and mammals (cattle SLC7A1 71.0%; mouse Slc7a1 69.9%; rhesus monkey and human SLC7A1 70.6%) (Fig. S2). Moreover, the alignment of the zebrafish Slc7a1a sequences revealed high identity percentage with respect to Slc7a1b sequences that varied between ~ 78 and ~ 69% depending on the teleost fish species analyzed (i.e., zebrafish Slc7a1b 77.5–77.8%; Atlantic herring Slc7a1b 72.3–72.4%; medaka Slc7a1b 74.1–75.2%; fugu rubripes Slc7a1b: 68.8–70.3%).

With respect to relevant protein motifs/regions, putative N-linked glycosylation sites conserved in mouse Slc7a1 and human SLC7A1 (Wang et al. 1996) in the third extracellular loop were—for example—also found in zebrafish Slc7a1a and the other teleost fish species studied (Fig. 1a, b; Fig. S1). Again, E^107^ of mouse Slc7a1, which has been shown to be necessary for the transport activity (Wang et al. 1994), was also conserved in zebrafish Slc7a1a and the other species compared (Fig. 1a and b; Fig. S1). Conversely, in teleost fish and amphibians, both the N- and C-terminal sequences were different than those found in mammals, and, e.g., an RRK motif near to the N-terminal that is conserved in higher vertebrates is replaced by RVK motif in the teleost fish species investigated in this study (Fig. S1).

Zebrafish *slc7a1a* localization on chromosome with respect to neighbor genes and in comparison to its orthologues along the vertebrate scale was evaluated by Gene analysis at NCBI, which clearly showed how *slc7a1a* (and not its paralogue *slc7a1b*) lays with *muts2a* within a syntenic region common to teleost fish, amphibians, birds and mammals (Table S3).

Taken together, all these gene/genomic findings strongly suggest the concept that zebrafish *slc7a1a* represents the true orthologue of the higher vertebrate *slc7a1*/*Slc7a1*/*SLC7A1* gene series.

### Specific expression during development

**Fig. 3:**
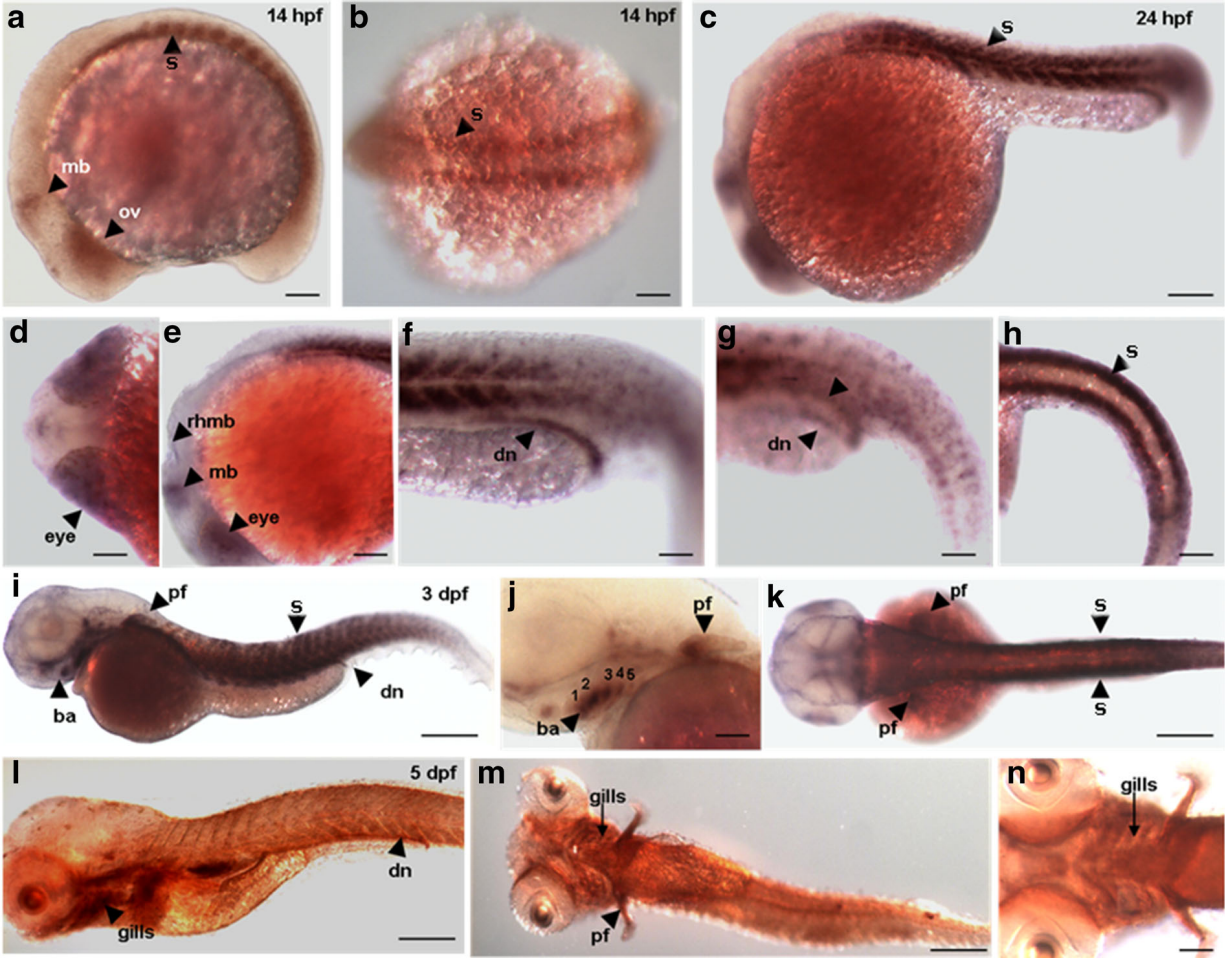
Spatiotemporal distribution of *slc7a1a* in zebrafish (*Danio rerio*). Image analysis after whole-mount *slc7a1a* in situ hybridization. **a** Lateral view shows expression of *slc7a1a* in optic vesicle, midbrain, and somites at 14 hpf. **b** Dorsal view shows *slc7a1a* expression in two stripes of somites at 14 hpf. **c** Lateral view of *slc7a1a* expression at 24 hpf. **d** Dorsal view of the expression in the eye at 24 hpf. **e** Lateral view shows the expression in the eye, posterior midbrain and dorsal edges of rhombomeres at 24 hpf. **f** Magnified lateral view showing expression in somites and distal nephron at 24 hpf. **g** Tilted lateral view shows two distal nephrons (arrows) fusing together at 24 hpf. **h** Dorsal view focusing on two strips of somites at 24 hpf. **i** Lateral view shows expression in branchial arches, pectoral fin, somites, and distal nephron at 3 dpf. **j** Magnified lateral view with gill arches and pectoral fin buds at 3 dpf. **k** Dorsal view showing expression in pectoral fins and somites at 3 dpf. **l** Lateral view at 5 dpf. **m** Ventral view 5 dpf. **n** Magnified view of gills at 5 dpf. Abbreviations: mb, midbrain; ov, optic vesicle; s, somites; dn, distal nephron, rhmb, rhombomeres; ba, branchial arches; pf, pectoral fin. Scale bar: **a**, **b**, **d**–**g**, **j**, **n**) 50 μm; **c**, **h**, **i**, **k**, **m**) 100 μm

Whole-mount in situ hybridization analysis of *slc7a1a* during embryonic development was used to analyze its expression at various stages of development ranging from 14 hpf to 5 dpf. This analysis first detected *slc7a1a* transcripts in somites, optic vesicles, and posterior midbrain around 14 hpf (Fig. 3a). Two distinct stripes (marked by arrows) of *slc7a1a* expression in somites were seen in a dorsal view at 14 hpf (Fig. 3b). More intense staining was observed at 24 hpf (Fig. 3c), with prominent expression in the eyes (Fig. 3d). Additional staining was detected in posterior midbrain, and a thin line of *slc7a1a* expression was observed along the dorsal edges of the rhombomeres at 24 hpf (Fig. 3e). Expression in the somites appeared as broad “v”-shaped stripes with no expression at the somite boundaries (Fig. 3f) that was also seen in a dorsal view as two distinct stripes parallel to the antero-posterior axis along the trunk and tail of the embryo (Fig. 3h). At the same stage, *slc7a1a* expression appeared in distal nephrons (Fig. 3f), which were clearly seen fusing together to form a common opening at the cloaca (arrowheads in Fig. 3g). This expression seems to correspond approximately to the distal late and pronephric duct segments (Wingert et al. 2007). In this respect, *slc7a1a* expression overlaps with both that of *slc12a3* in the distal late nephron and that of *gata3* in the pronephric duct segments (Wingert et al. 2007). Along with somites and distal nephrons, additional *slc7a1a* expression was observed in the pectoral fins and branchial arches at 3 dpf (Fig. 3i), while the expression in the eyes at this stage was almost disappeared. Upon close observation, five distinct branchial arches were seen marked by *slc7a1a* expression along with that of the pectoral fins (Fig. 3j). Somite expression was still maintained at 3 dpf (arrowheads in Fig. 3k). At 5 dpf, the *slc7a1a* expression was widespread in most parts of the embryo with stronger expression in the gills and distal nephrons (Fig. 3l). Five branchial arches forming gills were marked by stronger *slc7a1a* expression along with that in pectoral fins at 5 dpf (Fig. 3m, n).

## Discussion

Lysine and arginine are important amino acids in metabolism and nutrition. Lysine is essential in animals, i.e., it cannot be synthesized in the body and it needs to be obtained from an external food source. It is important for protein synthesis (growth) and carnitine production; also, it helps the body absorb calcium, and it is required for the formation of collagen, which is crucial for bones and connective tissues including skin, tendon, and cartilage (Civitelli et al. 1992; Flodin 1997; Fini et al. 2001). Arginine is essential for young mammals, while it is conditionally essential for adult mammals, since it is vital in situations such as pregnancy (Bronte and Zanovello 2005), spermatogenesis, maintenance of vascular tone, and hemodynamics (Wu et al. 2009). Arginine is also required for the detoxification of ammonia, which is highly toxic for the central nervous system (North et al. 2009; Wu et al. 2009). The vast majority of teleost fish including zebrafish are ammonotelic, excreting up to 90% of their nitrogenous waste directly into water as ammonia (Braun et al. 2009a, b).

Amino acid transporters are classified as to belong to the solute carrier (SLC) family of proteins, which consists of more than 400 organic and inorganic carriers (Hediger et al. 2013; Alexander et al. 2019). They transport amino acids over membranes and cells. A large array of amino acid transporters is present on the apical and basolateral membranes of intestinal epithelial cells, absorbing amino acids from the intestinal lumen for subsequent release into the blood (Broer 2008; Kandasamy et al. 2018). Amino acid transporters also have an important function in the vertebrate kidney where they are involved in adjustment of amino acid levels in the ultrafiltrate and final urine (Broer 2008; Kandasamy et al. 2018). In this study, we specifically focused on a zebrafish Cat-1 system, named Slc7a1a, which is putatively involved in the transport of lysine, arginine, and cysteine. By detailed sequence analysis, we conferred the structural and sequence specific identity of this zebrafish system, along with its syntenic conservation with evolutionarily related genes, and evidenced its expression during early development.

### Zebrafish Slc7a1a amino acid sequence is highly conserved

To determine identity and specificities of zebrafish Slc7a1a, we performed detailed sequence analysis. SLC7A1/Slc7a1 proteins from the different vertebrate species analyzed show a variable number of amino acids, e.g., mouse 622, human 629 (Deves and Boyd 1998), as well as rhesus monkey and cattle, chicken 624, while tropical clawed frog exhibited 654 amino acids. Conversely, teleost Slc7a1a proteins show the following amino acids lengths: zebrafish 650–651, Atlantic herring 656–657, medaka 647–648, and fugu rubripes 648, while teleost Slc7a1b proteins exhibit amino acids lengths as follows: zebrafish 646–647, Atlantic herring 654–655, medaka 638–639, and fugu rubripes from 638 to 681–682 depending on the isoform (for details, see Fig. S1). Therefore, zebrafish Slc7a1a and Slc7a1b proteins are more than 20 amino acids longer than mammalian SLC7A1/Slc7a1 proteins, and the length criterion is not a discriminating element to define orthology between SLC7A1/Slc7a1 and Slc7a1a or Slc7a1b transporters.

Analogously, SLC7A1/Slc7a1 proteins share similar percentage of identity with Slc7a1a and Slc7a1b proteins, e.g., with respect to human SLC7A1, these values span from ~ 71.5 to ~ 67.3% for Slc7a1a and from ~ 73.5 to ~ 66.8% for Slc7a1b transporters (for details, see Fig. S2). Therefore, also the identity criterion does not allow defining orthology between SLC7A1/Slc7a1 and SLc7a1a or Slc7a1b proteins.

Only additional comparison at the genomic level shows the (slight) syntenic conservation of the zebrafish, and teleost fish, *slc7a1a* gene with respect to the higher vertebrate *slc7a1*/*Slc7a1*/*SLC7A1* series. In fact, *slc7a1a* shares neighborhood with *mtus2a* in teleost fish while *slc7a1*/*Slc7a1*/*SLC7A1* genes in amphibians, birds, and mammals do with *mtus2*/*Mtus2*/*MTUS2*.

All together, these conserved features strongly support the authenticity of the predicted zebrafish Slc7a1a and put it in the orthologous line of the *slc7a1*/*Slc7a1*/*SLC7A1* series of higher vertebrates.

**Fig. 4:**
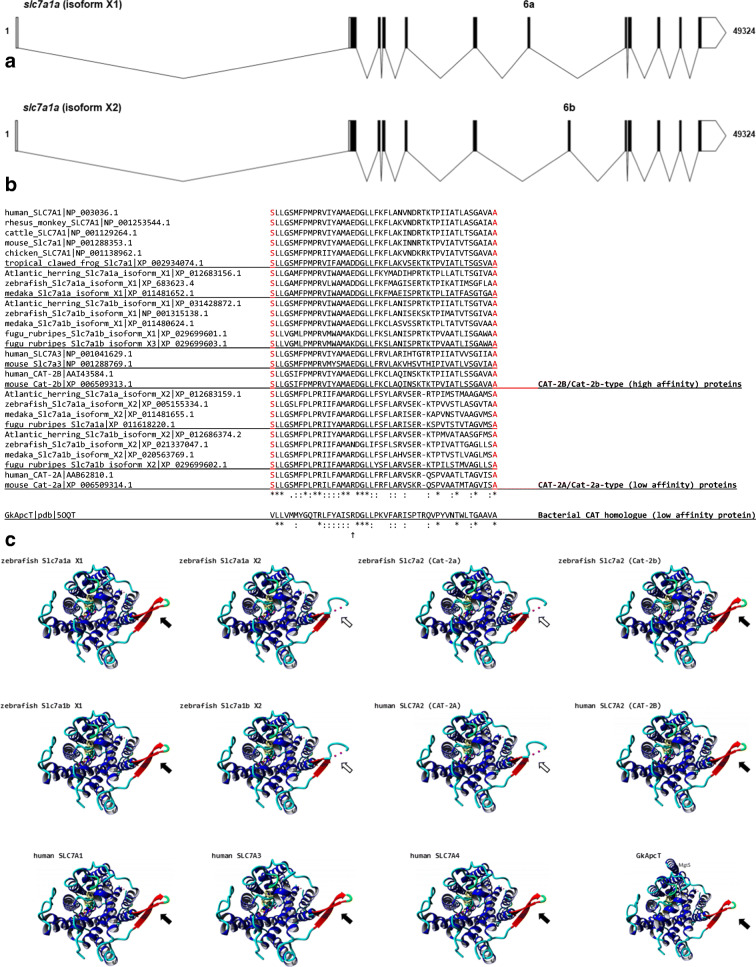
**a** Schematic diagram depicting the genomic (exon-intron) organization of the zebrafish *slc7a1a* gene. The zebrafish *slc7a1a* sequence is 49,324 nucleotides long. The alternative coding exons 6a and 6b are specifically indicated. Coding exon regions are in black, non-coding exon (5′- and 3′-untranslated) regions are in white (please note that a fully non-coding exon occurs upstream the zebrafish *slc7a1a* coding exon 1), and intron regions are as broken lines connecting exons. **b** Comparison of coding exon 6’s predicted amino acid sequences of teleost fish (Atlantic herring, zebrafish, medaka, fugu rubripes) Slc7a1a and Slc7a1b, amphibian (tropical clawed frog) Slc7a1, bird (chicken) SLC7A1, mammalian (human, rhesus monkey, cattle, mouse) Slc7a1/SLC7A1, mammalian (human, mouse) Slc7a2/SLC7A2, and mammalian (human, mouse) Slc7a3/SLC7A3 proteins. Multiple sequence alignment was generated using Clustal Omega at https://www.ebi.ac.uk/Tools/msa/clustalo/ using default parameters (for details, see also Fig. 1 and Fig. S1). GkApcT refers to the recently identified and characterized proton-coupled amino acid transporter with the leucine transporter (LeuT) fold from *Geobacillus kaustophilus* (Jungnickel et al. 2018; Wu et al. 2019). An upwards arrow marks an amino acid position within this region acknowledged to be relevant for the definition of the low vs. high substrate affinity state of the transporter. An arginine (R) associates with a low affinity state (see, e.g., Closs et al. 1997; Habermeier et al. 2003; Jungnickel et al. 2018; Wu et al. 2019). **c** Three-dimensional appearance (cytoplasmic view) of zebrafish Slc7a1a proteins (isoforms X1 and X2) and comparison with zebrafish Slc7a1b (isoforms X1 and X2) and Slc7a2 (Cat-2a and Cat-2b) and human SLC7A1, SLC7A2 (CAT-2A and CAT-2B), SLC7A3, and SLC7A4. Homology models as from Phyre^2^ (template: GkApcT) and visualization by YASARA (see also Fig. 1c). For each protein, confidence value was 100% and the residues in the model covered 70–71% of the corresponding experimental structure. The region of alternative splicing spans from approximately the second half of transmembrane domain 8 to the first half of transmembrane domain 9 through the cytoplasmic amino beta hairpin loop (see arrows). White arrows denote those proteins for which low substrate affinity has been ascertained (human CAT-2A) (see, e.g., Closs et al. 1997; Habermeier et al. 2003) or can be hypothesized (zebrafish Slc7a1a isoform X2, zebrafish Slc7a1b isoform X2 and zebrafish Cat-2a). MgtS indicates a small protein (31 amino acids; a single transmembrane domain) that complexes with GkApcT (Jungnickel et al. 2018; Wu et al. 2019)

Notably, beside the already mentioned E^107^ residue within the third TM domain, our sequence analysis identified a special feature represented by a set of conserved negatively and positively charged amino acids (e.g., R^362^, D^369^, D^370^, K^375^, E^382^, R^383^, T^384^, and K^385^) observable within a larger region encompassing the fourth intracellular loop in the zebrafish Slc7a1a isoform X1. This is substituted in isoform X2 by a similar stretch of amino acids but with differently charged amino acids in the parallel positions due to a splicing event involving exon 6a and exon 6b (for details, see Fig. 1 and 4 and Fig. S1). Such a feature is typical of both teleost fish Slc7a1a and Slc7a1b proteins. And this strongly brings such teleost fish protein types closer to the vertebrate SLC7A2/SLc7a2 proteins for the commonality of the splicing event that imparts the characteristics of high/low affinity toward cationic amino acids (and possibly sensitivity to *trans*-stimulation) to human SLC7A2 and mouse Slc7a2 (Closs et al. 1997; Habermeier et al. 2003). These findings are summarized in Fig. 4, which depicts the alternative splicing exons in the zebrafish *slc7a1a* gene structure (Fig. 4a), the alternative amino acid sequences in the zebrafish Slc7a1a isoforms X1 and X2 (Fig. 4b), and the structural differences that emerge between them by comparison after homology models analysis (Fig. 4c). Evident are, e.g., the differences in the structural organization of the hairpin loop between transmembrane domains 8 and 9, as evident is that the alternative sequences (Fig. 4b) and structures (Fig. 4c) of the zebrafish Slc7a1a proteins are typically shared by transporters for which low (i.e., mammalian CAT-2A) vs. high (i.e., mammalian CAT-2B) substrate affinity has been demonstrated. If confirmed functionally, a structural-functional paradigm so far attributed to SLC7A2/Slc7a2 proteins only would be to extend to other Cat-type proteins, such as the teleost fish Slc7a1a and Slc7a1b proteins.

Alignment also showed that the mammalian N-terminal peptide sequence motif (Met-Gly-Cys) is not conserved in the non-mammalian species. Downstream to this motif, there are several conserved basic residues along with two cysteines. In mouse, this conserved N-terminal motif along with the conserved basic residues of Slc7a1 has been shown to give a regulatory signal for transport into or retention inside of diverse cell membrane compartments (Ou and Silver 2003). Since this motif is absent but the downstream basic residues are conserved in teleost fish Slc7a1a (zebrafish included), an alternative regulatory signal might be present in these proteins.

### Slc7a1a expression in early eye, branchial arches, and muscle development might indicate a role in the collagen synthesis pathway

*slc7a1a* expression was observed in the developing eyes and somites as early as 14 hpf, which also continued at the 24-hpf stage. Although no visual function is present at 14 hpf, the Slc7a1-mediated arginine transport at the inner blood retinal barrier in rat has been indicated as relevant in visual functions since it provides precursors to NO in the neural retina (Tomi et al. 2009). Moreover, arginine is a precursor of collagen, which is the most abundant protein in animals (Berisio et al. 2002; Shoulders and Raines 2009) and its different types are associated with particular tissues such as skin, bone, tendon, ligaments, branchial arches, cornea, cartilage, kidney, glomeruli, retina, intestine, and more (Berisio et al. 2002; Shoulders and Raines 2009). Furthermore, biochemical collagen precursors such as proline or hydroxyproline derive from arginine, while the essential amino acid lysine also forms the majority of collagen (Wittmann et al. 2005; Barbul 2008). In this regard, the “putative-to-date” transport of arginine and lysine via Slc7a1a would act as a rate-limiting step in providing precursors for collagen synthesis. In addition, studies in zebrafish have reported various collagen types to be involved in development of morphological structures (Akhtar et al. 2008; Bader et al. 2009; Gansner and Gitlin 2008; Huang et al. 2009; Le Guellec et al. 2004; Pagnon-Minot et al. 2008; Xiao and Baier 2007). More specifically, TGF-*β*_1_ signaling has been implicated in collagen synthesis, where it stimulates proline and polyamine synthesis by upregulating arginine transport and metabolism mediated by a selective increase in *SLC7A1* mRNA, ornithine decarboxylase, and ornithine aminotransferase (Durante et al. 2001). These findings would support a hypothesis that *SLC7A1* might act in the collagen synthesis pathway required for the formation of eyes, branchial arches, and nephrons.

### Slc7a1a and kidney development

In zebrafish, two nephrons function as pronephric kidneys during early life stages, in contrast to the situation in mammalian species where the kidneys contain thousands of nephrons (Drummond 2003). The zebrafish pronephros contains 8 distinct segments including two proximal tubule segments and two distal tubule segments, which are similar to those of the mammalian nephron (Wingert et al. 2007; Wingert and Davidson 2008). Tubular fluid flow through pronephros starts as early as 24 hpf (Vasilyev et al. 2009), well before the formation of glomerulus, while glomerular filtration starts around 40 hpf (Drummond 2005; Vasilyev et al. 2009). The zebrafish pronephric nephrons form a closed system of blood filtration, tubular resorption, and fluid excretion (Drummond 2005). The primary function of the fish pronephros is osmoregulation (Drummond 2005). Without a functional kidney, zebrafish larvae die of gross edema since they are hyperosmotic animals that live in a very dilute environment (Drummond 2005). For instance, defects are reported in zebrafish morphants for *nephrin* and *podocin* (genes required for the development of pronephric podocyte cell structure) leading to defective proximal tubules and consequent pericardial edema (Kramer-Zucker et al. 2005).

In this study, *slc7a1a* expression was first observed in the distal part of nephrons at 24 hpf and was still maintained at 5 dpf. Previously, *SLC7A1* localization has been reported to the basolateral membrane of the polarized kidney epithelial (MDCK and HEK) cells (Kizhatil and Albritton 2002), while a decrease in its expression was observed during endothelial cell dysfunction related to chronic renal failure (Schwartz et al. 2006). In addition, arginine, that is the sole precursor for NO, governs NO synthesis in renal epithelial cells (Schwartz et al. 2008). Because of such a fundamental function, it could be hypothesized that a “still-to-be-demonstrated functionally” Slc7a1a-mediated arginine transport is relevant for the formation and function of zebrafish nephrons. In effects, studies in adult mice have shown that the bilateral ureteral ligation increases glomerular arginine transport via *Slc7a1* upregulation (Schwartz et al. 2008), while in chronic renal failure, arginine uptake is attenuated through modulation of *Slc7a1* (Schwartz et al. 2006).

### *slc7a1a* expression pattern indicates a role in ion regulation in the gills

*slc7a1a* expression started to appear in gill primordia at 3 dpf and appeared stronger at 5 dpf. This is consistent with the previous observation that the zebrafish pharyngeal arches produce gill filament primordia at 3 dpf (Kimmel et al. 1995; Rombough 2002). Ion regulation is the prime function of the gills before their gas-exchange and O_2_-chemosensory pathways develop (Rombough 2002), and in teleost gills, ion regulation is strictly associated with mitochondria-rich cell (MRC) located in the inter-lamellar region of the gill filament epithelium (Jonz and Nurse 2008). As a consequence, freshwater teleosts mediate Na^+^, Ca^2+^, and Cl^−^ uptake across the epithelium into the blood, while the saltwater teleosts mediate ion extrusion through MRCs (also called chloride cells) (Perry 1997; Marshall 2002; Evans et al. 2005; Jonz and Nurse 2008). Various neurotransmitters and neuropeptides, such as NO (eventually derived from arginine), catecholamines, acetylcholine, vasoactive intestinal polypeptide, endothelin, and prostaglandins, have been shown to mediate the movement of ions across gill and opercular epithelia, and stimulation of Cl^−^ transport is mediated by adrenergic receptors (Marshall 2002; Evans et al. 2004; Evans et al. 2005; Jonz and Nurse 2008). Considering this information, we conclude that Slc7a1a, being a source of arginine (precursor to NO), may be involved in gill ion regulation.

### Slc7a1a might be involved in ammonia excretion

It was originally considered that ammonia and urea move passively through tissues along partial pressure or concentration gradients. It is now proved that it requires Rhesus (Rh) proteins for ammonia transport (Marini et al. 1997) and urea transporter proteins (UT) for urea to efficiently cross plasma membranes (Levine et al. 1973; You et al. 1993; Shayakul et al. 1996; Braun et al. 2009b). We found *slc7a1a* to be expressed in the distal tubule segment of the nephron from 24 hpf and onwards, while it was expressed in the gills from 3 dpf. There is compelling evidence in animals (fish included) that, unlike storage of excess lipids and carbohydrate, the excess of amino acids and proteins is usually metabolized to ammonia (Wright 1995; Terjesen et al. 2002; Braun et al. 2009a). We propose that Slc7a1a-mediated transport may also be involved in the amino acid metabolism through the excretion of amino acid waste products such as ammonia and urea through kidney and gills. It has been reported that most teleost fishes excrete nitrogenous waste as ammonia through gills without converting it to urea or uric acid while freshwater fish can also excrete ammonia into urine to maintain the body fluid’s acid-base balance at low external pH (King and Goldstein 1983; Wood et al. 1999; Nakada et al. 2007). Notably, the ammonia transporter Rhcg1 was shown to be localized in apical MRCs of yolk sac, gill, and particularly in the distal tubules of the zebrafish nephron (Nakada et al. 2007). In addition, the knockdown of ammonia (Rhag, Rhbg, and Rhcg1) or urea transporters (UT) in developing zebrafish has been shown to reduce the ammonia and urea excretion (Braun et al. 2009a). Interestingly, the same study reported an expression of these ammonia and urea transporters in the gills and distal nephron of the 4-dpf zebrafish larvae (Braun et al. 2009a), which is similar to our observation of *slc7a1a* expression. Taken together, these findings suggest a possible link between the Slc7a1a (an amino acid transporter) and the Rh and UT (ammonia and urea transporter) in the amino acid metabolism. However, detailed co-localization and functional studies are essential to establish this link.

### Conclusion and perspectives

This study clarifies the gene/genomic organization of the zebrafish *slc7a1a* gene in the context of teleost fish Cat-type transporters and suggests the existence of a much more complex set up and regulation of Cat-type genes in zebrafish, and teleost fish genomes, with respect to higher vertebrates. Our study also shows the specific expression pattern of *slc7a1a* during zebrafish embryonic development suggesting the possible involvement in development and function of the eyes, kidney, muscles, and gills. The possible link between Slc7a1a-mediated cationic amino acid transport/homeostasis and ionoregulation and ammonia/urea transport, as well as other general physiological processes, needs to be established in zebrafish.

## Electronic supplementary material

ESM 1 (DOCX 96 kb)

## Data Availability

The authors confirm that the data supporting the findings of this study are available within the article and its supplementary materials.
