# Supplementary material for: Sequence analysis and spatiotemporal developmental distribution of the Cat-1-type transporter slc7a1a in zebrafish (Danio rerio)
Source: Fish Physiol Biochem. 2020 Sep 27;46(6):2281–98. doi: 10.1007/s10695-020-00873-x (PMC7584565; doi:10.1007/s10695-020-00873-x)
Supplement: Supplementary file 1 — (DOCX 96 kb) [file 10695_2020_873_MOESM1_ESM.docx]

**Fish Physiology and Biochemistry**

**Supplementary information**

**Sequence analysis and spatiotemporal developmental distribution of the Cat-1-type transporter *slc7a1a* in zebrafish (*Danio rerio*)**

Ståle Ellingsen ^1,3,4^, Shailesh Narawane ^1^, Anders Fjose ^1^, Tiziano Verri ^2^, Ivar Rønnestad ^3, *^

^1^ Department of Molecular Biology, University of Bergen, Postbox 7803, NO-5020 Bergen, Norway

^2^ Department of Biological and Environmental Sciences and Technologies, University of Salento, via Prov.le Lecce-Monteroni I-73100 Lecce, Italy

^3^ Department of Biological Sciences, University of Bergen, Postbox 7803, NO-5020 Bergen, Norway

**Table S1**

**Table S2**

**Table S3**

**Figure S1**

**Figure S2**

**Appendix I**

Table S1. Ensembl peptide IDs for *slc7a1*-related sequence used for analysis and comparison.

| **Species** | **Gene name** | **GenBank Acc. No.** | **Ensembl peptide ID** | **UniProtKB/UniParc peptide ID** |
| --- | --- | --- | --- | --- |
| Human | *SLC7A1* | NP_003036.1 | ENSP00000370128 | P30825 |
| Rhesus monkey | *SLC7A1* | NP_001253544.1 | ENSMMUP00000011357 | F7F495 |
| Mouse | *Slc7a1* | NP_001288353.1 | ENSMUSP00000046714 | Q09143 |
| Cattle | *SLC7A1* | NP_001129264.1 | ENSBTAP00000019060 | F1N5P6 |
| Chicken | *SLC7A1* | NP_001138962.1 | ENSGALP00000027546 | F1P388 |
| Tropical clawed frog | *slc7a1* | XP_002934074.1 | ENSXETP00000043883 | - |
| Zebrafish | *slc7a1a* (*slc7a1*) | XP_683623.4 (isoform X1)  XP_005155334.1 (isoform X2) | ENSDARP00000152628  ENSDARP00000027285 | A0A2R8QFJ7  E7F0D2 |
|  | *slc7a1b* (*zgc:63694*) | NP_001315138.1 (isoform X1)  XP_021337047.1 (isoform X2) | ENSDARP00000135330  - | A0A0G2KSE4  UPI000B442D4F |
| Atlantic herring | *slc7a1a* (*LOC105900401*) | XP_012683156.1 (isoform X1)  XP_012683159.1 (isoform X2) | ENSCHAP00000047785  ENSCHAP00000047888 | -  - |
|  | *slc7a1b* (*LOC105903209*) | XP_031428872.1 (isoform X1)  XP_012686374.2 (isoform X2) | -  - | -  - |
| Medaka | *slc7a1a* (*LOC101154797*) | XP_011481652.1 (isoform X1)  XP_011481655.1 (isoform X2) | ENSORLP00020027690  ENSORLP00020027669 | A0A3P9M4A0  A0A3P9M3Y3 |
|  | *slc7a1b* (*LOC101172427*) | XP_011480624.1 (isoform X1)  XP_020563769.1 (isoform X2) | -  - | UPI0002A47D84  UPI0005CBE6AC |
| Fugu rubripes | *slc7a1a* (*LOC101061314*) | XP_011618220.1 | - | - |
|  | *slc7a1b* (*LOC101070292*) | XP_029699601.1 (isoform X1)  XP_029699602.1 (isoform X2)  XP_029699603.1 (isoform X3) | -  -  - | UPI00114586C2  UPI0011460980  UPI001145A8E6 |

**Table S2.** Primers used for gene isolation and *in situ* probe preparation.

| **Gene symbol** | **Primer** | **Forward primer 5’🡪3’** | **Reverse primer 5’🡪3’** |
| --- | --- | --- | --- |
| *slc7a1a* | *in situ* probe | GCTGAGAGTAAAGGTTGTAAACTGC | GGACTGTTTTTATCCAGCATGTAAT |
|  | Full length cDNA | CAGCAATGGTTTTGAAAAAGCTTCTGC | gacattggttaaatcagtcatcgag |

**Table S3.** Localization of vertebrate *SLC7A1*, *Slc7a1*, *slc7a1*, *slc7a1a* and *slc7a1b* genes on their chromosomes and synteny relationships. Human, rhesus monkey, mouse, cattle, chicken, tropical clawed frog, zebrafish, Atlantic herring, medaka and fugu rubripes genes are highlighted (red arrows) and surrounded by the neighboring genes in their respective chromosomes. Results were obtained by Gene database consulting at the National Center for Biotechnology Information (<https://www.ncbi.nlm.nih.gov/gene>).

| **Species** | **Chromosome** | **Gene name** | **Gene position on chromosome** |
| --- | --- | --- | --- |
| **Human** | **Chr. 13 - NC_000013.11** | ***SLC7A1*** | 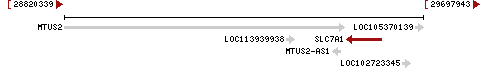 |
| **Rhesus monkey** | **Chr. 17 - NC_041770.1** | ***SLC7A1*** | 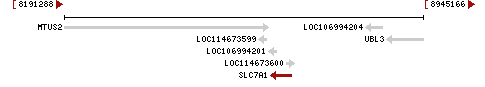 |
| **Cattle** | **Chr. 12 - NC_037339.1** | ***SLC7A1*** | 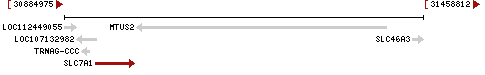 |
| **Mouse** | **Chr. 5 - NC_000071.6** | ***Slc7a1*** | 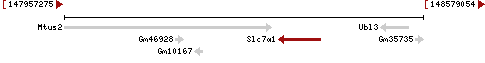 |
| **Chicken** | **Chr. 1 - NC_006088.5** | ***SLC7A1*** | 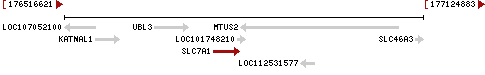 |
| **Tropical clawed frog** | **Chr. 2 - NC_030678.2** | ***slc7a1*** | 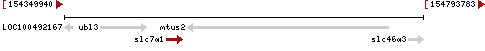 |
| **Zebrafish** | **Chr. 10 - NC_007121.7** | ***slc7a1a*** | 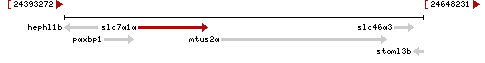 |
|  | **Chr. 15 - NC_007126.7** | ***slc7a1b*** | 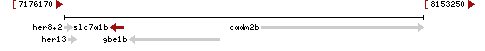 |
| **Atlantic herring** | **Chr. 8 - NC_045159.1** | ***slc7a1a*** | 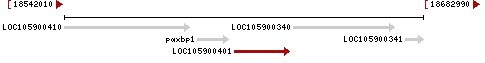 |
|  | **Chr. 9 - NC_045160.1** | ***slc7a1b*** | 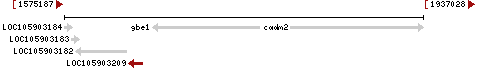 |
| **Medaka** | **Chr. 14 - NC_019872.2** | ***slc7a1a*** | 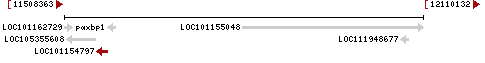 |
|  | **Chr. 13 - NC_019871.2** | ***slc7a1b*** | 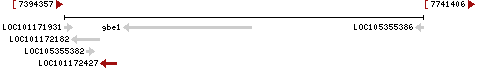 |
| **Fugu** | **Chr. 15 - NC_042299.1** | ***slc7a1a*** | 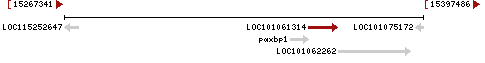 |
|  | **Chr. 11 - NC_042295.1** | ***slc7a1b*** | 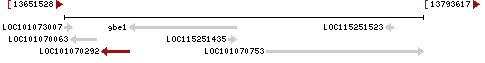 |

*cadm2*, cell adhesion molecule 2; *cadm2b*, cell adhesion molecule 2b; *gbe1*, glucan (1,4-alpha-), branching enzyme 1; *gbe1b*, glucan (1,4-alpha-), branching enzyme 1b; *Gm*, predicted gene or pseudogene; *hephl1b*, hephaestin-like 1b; *her13*, hairy-related 13; *her8.2*, hairy-related 8.2; *KATNAL1*, katanin catalytic subunit A1 like 1; *LOC*, locus; *MTUS2* or *Mtus2* or *mtus2*, microtubule associated scaffold protein 2; *mtus2a*, microtubule associated tumor suppressor candidate 2a; *MTUS2-AS1*, MTUS2 antisense RNA 1; *paxbp1*, PAX3 and PAX7 binding protein 1; *SLC46A3* or *slc46a3*, solute carrier family 46 member 3; *stoml3b*, stomatin (EPB72)-like 3b; *TRNAG-CCC*, transfer RNA glycine (anticodon CCC); *UBL3* or *Ubl3* or *ubl3*, ubiquitin like 3.

human_SLC7A1|NP_003036.1 -----------------------------------------------MGCKVLLNIGQQM 13

rhesus_monkey_SLC7A1|NP_001253544.1 -----------------------------------------------MGCKVLLNIGQQM 13

cattle_SLC7A1|NP_001129264.1 -----------------------------------------------MGCTMLKGVGQQM 13

mouse_Slc7a1|NP_001288353.1 -----------------------------------------------MGCKNLLGLGQQM 13

chicken_SLC7A1|NP_001138962.1 -----------------------------------------------MECQKFIKFGNQL 13

tropical_clawed_frog_Slc7a1|XP_002934074.1 ----------------------------------------------MNSGDILLTFGQQL 14

Atlantic_herring_Slc7a1a_isoform_X1|XP_012683156.1 -----------------------------------------------MVLHKLLDFGKQL 13

Atlantic_herring_Slc7a1a_isoform_X2|XP_012683159.1 -----------------------------------------------MVLHKLLDFGKQL 13

zebrafish_Slc7a1a_isoform_X1|XP_683623.4 -----------------------------------------------MVLKKLLRFGKQL 13

zebrafish_Slc7a1a_isoform_X2|XP_005155334.1 -----------------------------------------------MVLKKLLRFGKQL 13

medaka_Slc7a1a_isoform_X1|XP_011481652.1 -----------------------------------------------MALQALIHFGKQL 13

medaka_Slc7a1a_isoform_X2|XP_011481655.1 -----------------------------------------------MALQALIHFGKQL 13

fugu_rubripes_Slc7a1a|XP_011618220.1 -----------------------------------------------MALKRLLGFGKQL 13

Atlantic_herring_Slc7a1b_isoform_X1|XP_031428872.1 -----------------------------------------------MFLSTLKDLGRQL 13

Atlantic_herring_Slc7a1b_isoform_X2|XP_012686374.2 -----------------------------------------------MFLSTLKDLGRQL 13

zebrafish_Slc7a1b_isoform_X1|NP_001315138.1 -----------------------------------------------MVLTIMKELGKQL 13

zebrafish_Slc7a1b_isoform_X2|XP_021337047.1 -----------------------------------------------MVLTIMKELGKQL 13

medaka_Slc7a1b_isoform_X1|XP_011480624.1 -----------------------------------------------MLLATLKGLGRKL 13

medaka_Slc7a1b_isoform_X2|XP_020563769.1 -----------------------------------------------MLLATLKGLGRKL 13

fugu_rubripes_Slc7a1b_isoform_X1|XP_029699601.1 MNQSGNCSRDTTAAGKVGFKPEKRDRSLWCPRFLAVVFLLSGAKMRIKMLATLKDIGSRL 60

fugu_rubripes_Slc7a1b_isoform_X2|XP_029699602.1 MNQSGNCSRDTTAAGKVGFKPEKRDRSLWCPRFLAVVFLLSGAKMRIKMLATLKDIGSRL 60

fugu_rubripes_Slc7a1b_isoform_X3|XP_029699603.1 --------------------------------------------MRIKMLATLKDIGSRL 16

: .* ::

**<----------1----------> <----------**

human_SLC7A1|NP_003036.1 LRRKVVDCSREETRLSRCLNTFDLVALGVGSTLGAGVYVLAGAVARENAGPAIVISFLIA 73

rhesus_monkey_SLC7A1|NP_001253544.1 LRRKVVDCSREETRLSRCLNTFDLVALGVGSTLGAGVYVLAGAVARENAGPAIVISFLIA 73

cattle_SLC7A1|NP_001129264.1 LRRKVVDCSREESRLSRCLNTFDLVALGVGSTLGAGVYVLAGAVARENAGPAIVISFLIA 73

mouse_Slc7a1|NP_001288353.1 LRRKVVDCSREESRLSRCLNTYDLVALGVGSTLGAGVYVLAGAVARENAGPAIVISFLIA 73

chicken_SLC7A1|NP_001138962.1 LRRKNVDCTREDSRLSRCLNTFDLVALGVGSTLGAGVYVLAGAVARVNAGPAIVISFLIA 73

tropical_clawed_frog_Slc7a1|XP_002934074.1 FRRKVVGSDSKESRLSRCLNTFDLVALGVGSTLGAGVYVLAGAVARENAGPAIVLCFLIA 74

Atlantic_herring_Slc7a1a_isoform_X1|XP_012683156.1 LRVKVVDCSIEDSRLSRCLNTFDLVALGVGSTLGAGVYVLAGAVARESAGPAIVLSFLIA 73

Atlantic_herring_Slc7a1a_isoform_X2|XP_012683159.1 LRVKVVDCSIEDSRLSRCLNTFDLVALGVGSTLGAGVYVLAGAVARESAGPAIVLSFLIA 73

zebrafish_Slc7a1a_isoform_X1|XP_683623.4 LRVKVVNCNSEESRLSRCLNTFDLVALGVGSTLGAGVYVLAGAVARENAGPAIVLSFLIA 73

zebrafish_Slc7a1a_isoform_X2|XP_005155334.1 LRVKVVNCNSEESRLSRCLNTFDLVALGVGSTLGAGVYVLAGAVARENAGPAIVLSFLIA 73

medaka_Slc7a1a_isoform_X1|XP_011481652.1 LRVKVVNGNSEDSRLSRCLDTFDLVALGVGSTLGAGVYVLAGAVARDNSGPAIVLSFLIA 73

medaka_Slc7a1a_isoform_X2|XP_011481655.1 LRVKVVNGNSEDSRLSRCLDTFDLVALGVGSTLGAGVYVLAGAVARDNSGPAIVLSFLIA 73

fugu_rubripes_Slc7a1a|XP_011618220.1 LRVKVVDCNSEESHLSRCLNTFDLVALGVGSTLGAGVYVLAGAVARENSGPAIVLCFLIA 73

Atlantic_herring_Slc7a1b_isoform_X1|XP_031428872.1 LRVKVVDCSATESRLARCLNTFDLVALGVGSTLGAGVYVLAGAVARENSGPAIVLSFLIA 73

Atlantic_herring_Slc7a1b_isoform_X2|XP_012686374.2 LRVKVVDCSATESRLARCLNTFDLVALGVGSTLGAGVYVLAGAVARENSGPAIVLSFLIA 73

zebrafish_Slc7a1b_isoform_X1|NP_001315138.1 LRVKVVDCSTEESRLSRCLNTFDLVALGVGSTLGAGVYVLAGAVARENAGPAIVLSFLIA 73

zebrafish_Slc7a1b_isoform_X2|XP_021337047.1 LRVKVVDCSTEESRLSRCLNTFDLVALGVGSTLGAGVYVLAGAVARENAGPAIVLSFLIA 73

medaka_Slc7a1b_isoform_X1|XP_011480624.1 LRVKVVDCSTEESRLSRCLGTFDLVALGVGSTLGAGVYVLAGAVARENSGPAIVLSFLIA 73

medaka_Slc7a1b_isoform_X2|XP_020563769.1 LRVKVVDCSTEESRLSRCLGTFDLVALGVGSTLGAGVYVLAGAVARENSGPAIVLSFLIA 73

fugu_rubripes_Slc7a1b_isoform_X1|XP_029699601.1 LRVKVMDCSTEESHFSRCLNTFDLVALGVGSTLGAGVYVLAGAVARDTSGPAIVLSFFIA 120

fugu_rubripes_Slc7a1b_isoform_X2|XP_029699602.1 LRVKVMDCSTEESHFSRCLNTFDLVALGVGSTLGAGVYVLAGAVARDTSGPAIVLSFFIA 120

fugu_rubripes_Slc7a1b_isoform_X3|XP_029699603.1 LRVKVMDCSTEESHFSRCLNTFDLVALGVGSTLGAGVYVLAGAVARDTSGPAIVLSFFIA 76

:* * :. :::::***.*:************************ .:*****:.*:**

**-2---------> <----------3----------->**

human_SLC7A1|NP_003036.1 ALASVLAGLCYGEFGARVPKTGSAYLYSYVTVGELWAFITGWNLILSYIIGTSSVARAWS 133

rhesus_monkey_SLC7A1|NP_001253544.1 ALASVLAGLCYGEFGARVPKTGSAYLYSYVTVGELWAFITGWNLILSYIIGTSSVARAWS 133

cattle_SLC7A1|NP_001129264.1 ALASVLAGLCYGEFGARVPKTGSAYLYSYVTVGELWAFITGWNLILSYIIGTSSVARAWS 133

mouse_Slc7a1|NP_001288353.1 ALASVLAGLCYGEFGARVPKTGSAYLYSYVTVGELWAFITGWNLILSYIIGTSSVARAWS 133

chicken_SLC7A1|NP_001138962.1 ALASVLAGLCYGEFGARVPKTGSAYLYSYVTVGELWAFITGWNLILSYVIGTSSVARAWS 133

tropical_clawed_frog_Slc7a1|XP_002934074.1 ALASVLAGLCYGEFGARVPKTGSAYLYSYVTVGEFLAFVTGWNLILSYVIGTSSVARAWS 134

Atlantic_herring_Slc7a1a_isoform_X1|XP_012683156.1 ALASVMAGLCYAEFGARVPKTGSAYLYSYVTVGELWAFITGWNLILSYVIGTSSVARAWS 133

Atlantic_herring_Slc7a1a_isoform_X2|XP_012683159.1 ALASVMAGLCYAEFGARVPKTGSAYLYSYVTVGELWAFITGWNLILSYVIGTSSVARAWS 133

zebrafish_Slc7a1a_isoform_X1|XP_683623.4 ALASVLAGLCYAEFGARVPKTGSAYLYSYVTVGELWAFITGWNLILSYVIGTSSVARAWS 133

zebrafish_Slc7a1a_isoform_X2|XP_005155334.1 ALASVLAGLCYAEFGARVPKTGSAYLYSYVTVGELWAFITGWNLILSYVIGTSSVARAWS 133

medaka_Slc7a1a_isoform_X1|XP_011481652.1 ASASVLAGLCYAEFGARVPKTGSAYLYSYVTVGELWAFITGWNLILSYIIGTSSVARAWS 133

medaka_Slc7a1a_isoform_X2|XP_011481655.1 ASASVLAGLCYAEFGARVPKTGSAYLYSYVTVGELWAFITGWNLILSYIIGTSSVARAWS 133

fugu_rubripes_Slc7a1a|XP_011618220.1 ALASVLAGLCYAEFGARVPKTGSAYLYSYVTVGEIWAFFTGWNLILSYVIGTSSVARAWS 133

Atlantic_herring_Slc7a1b_isoform_X1|XP_031428872.1 ALASVMAGLCYAEFGARVPKTGSAYLYSYVTVGELWAFITGWNLILSYIIGTSSVARAWS 133

Atlantic_herring_Slc7a1b_isoform_X2|XP_012686374.2 ALASVMAGLCYAEFGARVPKTGSAYLYSYVTVGELWAFITGWNLILSYIIGTSSVARAWS 133

zebrafish_Slc7a1b_isoform_X1|NP_001315138.1 ALASVMAGLCYAEFGARVPRTGSAYLYSYVTVGELWAFITGWNLILSYIIGTSSVARAWS 133

zebrafish_Slc7a1b_isoform_X2|XP_021337047.1 ALASVMAGLCYAEFGARVPRTGSAYLYSYVTVGELWAFITGWNLILSYIIGTSSVARAWS 133

medaka_Slc7a1b_isoform_X1|XP_011480624.1 ALASVLAGLCYAEFGARVPRTGSAYLYSYVTVGELWAFITGWNLILSYVIGTSSVARAWS 133

medaka_Slc7a1b_isoform_X2|XP_020563769.1 ALASVLAGLCYAEFGARVPRTGSAYLYSYVTVGELWAFITGWNLILSYVIGTSSVARAWS 133

fugu_rubripes_Slc7a1b_isoform_X1|XP_029699601.1 ALASVLAGLCYAEFGARVPKTGSAYLYTYVTVGELWAFITGWNLILSYVIGTASVARAWS 180

fugu_rubripes_Slc7a1b_isoform_X2|XP_029699602.1 ALASVLAGLCYAEFGARVPKTGSAYLYTYVTVGELWAFITGWNLILSYVIGTASVARAWS 180

fugu_rubripes_Slc7a1b_isoform_X3|XP_029699603.1 ALASVLAGLCYAEFGARVPKTGSAYLYTYVTVGELWAFITGWNLILSYVIGTASVARAWS 136

* ***:*****.*******:*******:******: **.*********:***:*******

**<-------------4---------> <-----**

human_SLC7A1|NP_003036.1 ATFDELIGRPIGEFSRTHMTLNAPGVLAENPDIFAVIIILILTGLLTLGVKESAMVNKIF 193

rhesus_monkey_SLC7A1|NP_001253544.1 ATFDELIGRPIGEFSRTHMALNAPGVLAENPDIFAVIIILILTGLLTLGVKESAMVNKIF 193

cattle_SLC7A1|NP_001129264.1 ATFDELIGKPIGEFSRTHMALHAPGVLAENPDIFAVIIIVILTGLLTLGVKESAMVNKIF 193

mouse_Slc7a1|NP_001288353.1 ATFDELIGKPIGEFSRQHMALNAPGVLAQTPDIFAVIIIIILTGLLTLGVKESAMVNKIF 193

chicken_SLC7A1|NP_001138962.1 ATFDEIIGGHIEDFCKRYMTMNAPGVLAKYPDIFAVVIIIILTGLLTFGVKESALVNKVF 193

tropical_clawed_frog_Slc7a1|XP_002934074.1 ATFDDLIGKRIEEYCLAHISMNFPGVLARYPDIFSVLIILVLTGLLSFGVKESALVNKVF 194

Atlantic_herring_Slc7a1a_isoform_X1|XP_012683156.1 ATFDELIGKHIEAFCRLNMPMNAPGILAEYPDMFAVLIIITLTGLLSFGVKESAVVNKVF 193

Atlantic_herring_Slc7a1a_isoform_X2|XP_012683159.1 ATFDELIGKHIEAFCRLNMPMNAPGILAEYPDMFAVLIIITLTGLLSFGVKESAVVNKVF 193

zebrafish_Slc7a1a_isoform_X1|XP_683623.4 ATFDELIGKHIEHFCRQYMSMNAPGVLAEYPDMFSVFIILTLTGLLAFGVKESAMVNKVF 193

zebrafish_Slc7a1a_isoform_X2|XP_005155334.1 ATFDELIGKHIEHFCRQYMSMNAPGVLAEYPDMFSVFIILTLTGLLAFGVKESAMVNKVF 193

medaka_Slc7a1a_isoform_X1|XP_011481652.1 ATFDELIGKHIEQFCRTYMSMSAPGVLAEYPDMFAVFIIFTLTGLLAFGVKESAMVNKVF 193

medaka_Slc7a1a_isoform_X2|XP_011481655.1 ATFDELIGKHIEQFCRTYMSMSAPGVLAEYPDMFAVFIIFTLTGLLAFGVKESAMVNKVF 193

fugu_rubripes_Slc7a1a|XP_011618220.1 ATFDELIGKHIEHFCRAYMSMNAPGVLAEYPDAFAVVIIITLTGLLAFGVKESAMVNKVF 193

Atlantic_herring_Slc7a1b_isoform_X1|XP_031428872.1 ATFDGLIGNHIEKFCKQYMNMKAEGILAEYPDMFAVLIIITLTGLLAFGVKESAMVNKVF 193

Atlantic_herring_Slc7a1b_isoform_X2|XP_012686374.2 ATFDGLIGNHIEKFCKQYMNMKAEGILAEYPDMFAVLIIITLTGLLAFGVKESAMVNKVF 193

zebrafish_Slc7a1b_isoform_X1|NP_001315138.1 ATFDELIGKHIEEFCRRHMSINTPG-LAEYPDMFAVIIILTLTGLLAFGVKESAMVNKVF 192

zebrafish_Slc7a1b_isoform_X2|XP_021337047.1 ATFDELIGKHIEEFCRRHMSINTPG-LAEYPDMFAVIIILTLTGLLAFGVKESAMVNKVF 192

medaka_Slc7a1b_isoform_X1|XP_011480624.1 ATFDELINGHIKRFSSQYLPMNAPGV-AEYPDIFAVLIILSLTGLLVFGVKESALVNKVF 192

medaka_Slc7a1b_isoform_X2|XP_020563769.1 ATFDELINGHIKRFSSQYLPMNAPGV-AEYPDIFAVLIILSLTGLLVFGVKESALVNKVF 192

fugu_rubripes_Slc7a1b_isoform_X1|XP_029699601.1 ATFDKMIGKYIEEFCRQHMTMDAPGVLAEYPDIFAVFIIIILTGLLAFGVKESAWVNKVF 240

fugu_rubripes_Slc7a1b_isoform_X2|XP_029699602.1 ATFDKMIGKYIEEFCRQHMTMDAPGVLAEYPDIFAVFIIIILTGLLAFGVKESAWVNKVF 240

fugu_rubripes_Slc7a1b_isoform_X3|XP_029699603.1 ATFDKMIGKYIEEFCRQHMTMDAPGVLAEYPDIFAVFIIIILTGLLAFGVKESAWVNKVF 196

**** :*. * :. : : * *. ** *:*.**. ***** :****** ***:*

**-----5----------> <--**

human_SLC7A1|NP_003036.1 TCINVLVLGFIMVSGFVKGSVKNWQLTEEDFGNTSGR-----LCLNN-DTKEGKPGVGGF 247

rhesus_monkey_SLC7A1|NP_001253544.1 TCVNVLVLGFIMVSGFVKGSVKNWQLTEEDFGNTSGR-----LCLNN-DTKEGKPGVGGF 247

cattle_SLC7A1|NP_001129264.1 TCINVLVLGFIMVSGFVKGSIKNWQLTEEDFRNTSGH-----LCLNN-DTKEGKPGVGGF 247

mouse_Slc7a1|NP_001288353.1 TCINVLVLCFIVVSGFVKGSIKNWQLTEKNFSC------------NN-NDTNVKYGEGGF 240

chicken_SLC7A1|NP_001138962.1 TCINILVIGFVVVSGFVKGSVKNWQLTERDIYNTSPG-----IHGDNQTQGEKLYGVGGF 248

tropical_clawed_frog_Slc7a1|XP_002934074.1 TCINVLVLGFVIISGFVKGSVKNWQLSENDFNVTNVT-----GLPNGTKEQDLNYGAGGF 249

Atlantic_herring_Slc7a1a_isoform_X1|XP_012683156.1 TCINVLVLVFMVVSGLVKGTLKNWQLVPEEILNGT----NSSLNGTDPALVGVDLGVGGF 249

Atlantic_herring_Slc7a1a_isoform_X2|XP_012683159.1 TCINVLVLVFMVVSGLVKGTLKNWQLVPEEILNGT----NSSLNGTDPALVGVDLGVGGF 249

zebrafish_Slc7a1a_isoform_X1|XP_683623.4 TCINILVLLFMVVSGLVKGTLKNWHLDPDEILNAT----NSTLNATQPLPSEEMLGQGGF 249

zebrafish_Slc7a1a_isoform_X2|XP_005155334.1 TCINILVLLFMVVSGLVKGTLKNWHLDPDEILNAT----NSTLNATQPLPSEEMLGQGGF 249

medaka_Slc7a1a_isoform_X1|XP_011481652.1 TCINVLVLLFMVVSGLVKGTIKNWQINPEEILNGTF-TSNSSQNQTEVLPTKEILGAGGF 252

medaka_Slc7a1a_isoform_X2|XP_011481655.1 TCINVLVLLFMVVSGLVKGTIKNWQINPEEILNGTF-TSNSSQNQTEVLPTKEILGAGGF 252

fugu_rubripes_Slc7a1a|XP_011618220.1 TCINVLVLLFMVISGLVKGTMKNWQIDPEEILKANHTTSNSSLNLTELLPSRESMGAGGF 253

Atlantic_herring_Slc7a1b_isoform_X1|XP_031428872.1 TCVNVLVLLFVVVSGLVKGTLKNWNIDPEEVLHYNG---SGTYNLSETLPTPAEVGAGGF 250

Atlantic_herring_Slc7a1b_isoform_X2|XP_012686374.2 TCVNVLVLLFVVVSGLVKGTLKNWNIDPEEVLHYNG---SGTYNLSETLPTPAEVGAGGF 250

zebrafish_Slc7a1b_isoform_X1|NP_001315138.1 TCVNVLVLMFVVVSGLIKGTLQNWNLNPEDILNHTT---S--ANVSVPLPTEESLGFGGF 247

zebrafish_Slc7a1b_isoform_X2|XP_021337047.1 TCVNVLVLMFVVVSGLIKGTLQNWNLNPEDILNHTT---S--ANVSVPLPTEESLGFGGF 247

medaka_Slc7a1b_isoform_X1|XP_011480624.1 TCINVLVLLFVIIAGFVKGDRKNWSLNPEDILNSTR---NSTLNTTASLASEETIGAGGF 249

medaka_Slc7a1b_isoform_X2|XP_020563769.1 TCINVLVLLFVIIAGFVKGDRKNWSLNPEDILNSTR---NSTLNTTASLASEETIGAGGF 249

fugu_rubripes_Slc7a1b_isoform_X1|XP_029699601.1 TSVNVVVLVFVIISGFVKGNLKNWSLNPEEIFNSTS---NSSLNLSSPALSEDVLGAGGF 297

fugu_rubripes_Slc7a1b_isoform_X2|XP_029699602.1 TSVNVVVLVFVIISGFVKGNLKNWSLNPEEIFNSTS---NSSLNLSSPALSEDVLGAGGF 297

fugu_rubripes_Slc7a1b_isoform_X3|XP_029699603.1 TSVNVVVLVFVIISGFVKGNLKNWSLNPEEIFNSTS---NSSLNLSSPALSEDVLGAGGF 253

*.:*::*: *::::*::** :** : :. * ***

**---------6-----------> <-----------7-------**

human_SLC7A1|NP_003036.1 MPFGFSGVLSGAATCFYAFVGFDCIATTGEEVKNPQKAIPVGIVASLLICFIAYFGVSAA 307

rhesus_monkey_SLC7A1|NP_001253544.1 MPFGFSGVLSGAATCFYAFVGFDCIATTGEEVKNPQKAIPVGIVASLLICFIAYFGVSAA 307

cattle_SLC7A1|NP_001129264.1 MPFGFSGVLSGAATCFYAFVGFDCIATTGEEVKNPQKAIPVGIVASLLICFIAYFGVSAA 307

mouse_Slc7a1|NP_001288353.1 MPFGFSGVLSGAATCFYAFVGFDCIATTGEEVKNPQKAIPVGIVASLLICFIAYFGVSAA 300

chicken_SLC7A1|NP_001138962.1 MPYGLKGVLSGAATCFYAFVGFDCIATTGEEVKNPQKAIPIGIVASLLICFVAYFGVSAA 308

tropical_clawed_frog_Slc7a1|XP_002934074.1 MPFGFAGVLSGAATCFYAFVGFDCIATTGEEVKNPQKAIPIGIVASLLICFVAYFGVSAA 309

Atlantic_herring_Slc7a1a_isoform_X1|XP_012683156.1 MPYGFTGVLSGAATCFYAFVGFDCIATTGEEVKNPQRAIPIGIVSSLLICFVAYFGVSAA 309

Atlantic_herring_Slc7a1a_isoform_X2|XP_012683159.1 MPYGFTGVLSGAATCFYAFVGFDCIATTGEEVKNPQRAIPIGIVSSLLICFVAYFGVSAA 309

zebrafish_Slc7a1a_isoform_X1|XP_683623.4 MPFGFTGVLSGAATCFYAFVGFDCIATTGEEVKNPQRAIPIGIVSSLLICFVAYFGVSAA 309

zebrafish_Slc7a1a_isoform_X2|XP_005155334.1 MPFGFTGVLSGAATCFYAFVGFDCIATTGEEVKNPQRAIPIGIVSSLLICFVAYFGVSAA 309

medaka_Slc7a1a_isoform_X1|XP_011481652.1 MPFGFTGVLSGAATCFYAFVGFDCIATTGEEVKNPQRAIPIGIVSSLLICFVAYFGVSAA 312

medaka_Slc7a1a_isoform_X2|XP_011481655.1 MPFGFTGVLSGAATCFYAFVGFDCIATTGEEVKNPQRAIPIGIVSSLLICFVAYFGVSAA 312

fugu_rubripes_Slc7a1a|XP_011618220.1 MPFGWSGVLSGAATCFYAFVGFDCIATTGEEVKNPQRAIPIGIVSSLLICFVAYFGVSAA 313

Atlantic_herring_Slc7a1b_isoform_X1|XP_031428872.1 MPFGVKGVLSGAATCFYAFVGFDCIATTGEEVKNPQRAIPIGIVASLLICFLAYFGVSAA 310

Atlantic_herring_Slc7a1b_isoform_X2|XP_012686374.2 MPFGVKGVLSGAATCFYAFVGFDCIATTGEEVKNPQRAIPIGIVASLLICFLAYFGVSAA 310

zebrafish_Slc7a1b_isoform_X1|NP_001315138.1 MPFGFSGVLSGAATCFYAFVGFDCIATTGEEVKNPQRAIPIGIVASLLICFVAYFGVSAA 307

zebrafish_Slc7a1b_isoform_X2|XP_021337047.1 MPFGFSGVLSGAATCFYAFVGFDCIATTGEEVKNPQRAIPIGIVASLLICFVAYFGVSAA 307

medaka_Slc7a1b_isoform_X1|XP_011480624.1 MPFGWTGVLSGAATCFYAFVGFDCIATTGEEVKNPQRAIPIGIVASLLICFVAYFGVSAA 309

medaka_Slc7a1b_isoform_X2|XP_020563769.1 MPFGWTGVLSGAATCFYAFVGFDCIATTGEEVKNPQRAIPIGIVASLLICFVAYFGVSAA 309

fugu_rubripes_Slc7a1b_isoform_X1|XP_029699601.1 MPFGWSGVLSGAATCFYAFIGFDCIATTGEEVKNPQRAIPVGIVASLLICFVAYFGVSAA 357

fugu_rubripes_Slc7a1b_isoform_X2|XP_029699602.1 MPFGWSGVLSGAATCFYAFIGFDCIATTGEEVKNPQRAIPVGIVASLLICFVAYFGVSAA 357

fugu_rubripes_Slc7a1b_isoform_X3|XP_029699603.1 MPFGWSGVLSGAATCFYAFIGFDCIATTGEEVKNPQRAIPVGIVASLLICFVAYFGVSAA 313

**:* *************:****************:***:***:******:********

**----> <----------------8------------------->**

human_SLC7A1|NP_003036.1 LTLMMPYFCLDNNSPLPDAFKHVGWEGAKYAVAVGSLCALSASLLGSMFPMPRVIYAMAE 367

rhesus_monkey_SLC7A1|NP_001253544.1 LTLMMPYFCLDNNSPLPDAFKHVGWEGAKYAVAVGSLCALSASLLGSMFPMPRVIYAMAE 367

cattle_SLC7A1|NP_001129264.1 LTLMMPYFCLDKDSPLPDAFKHVGWEGAKYAVAVGSLCALSTSLLGSMFPMPRVIYAMAE 367

mouse_Slc7a1|NP_001288353.1 LTLMMPYFCLDIDSPLPGAFKHQGWEEAKYAVAIGSLCALSTSLLGSMFPMPRVIYAMAE 360

chicken_SLC7A1|NP_001138962.1 LTLMMPYYQLDTNSPLPNAFKYVGWDGANYAVAVGSLCALSTSLLGSMFPMPRIIYAMAE 368

tropical_clawed_frog_Slc7a1|XP_002934074.1 LTLMMPYYLLNKESPLPVAFNYVGWDGARYAVAVGSLCALSTSLLGSMFPMPRVIFAMAD 369

Atlantic_herring_Slc7a1a_isoform_X1|XP_012683156.1 LTMMMPYYMLDKNSPLPVAFKYVGWEGATYAVAVGSLCALSTSLLGAMFPMPRVIWAMAE 369

Atlantic_herring_Slc7a1a_isoform_X2|XP_012683159.1 LTMMMPYYMLDKNSPLPVAFKYVGWEGATYAVAVGSLCALSTSLLGSMFPLPRIIFAMAR 369

zebrafish_Slc7a1a_isoform_X1|XP_683623.4 LTMMMPYYMLDKNSPLPVAFKYVGWEGATYAVAVGSLCALSTSLLGAMFPMPRVLWAMAD 369

zebrafish_Slc7a1a_isoform_X2|XP_005155334.1 LTMMMPYYMLDKNSPLPVAFKYVGWEGATYAVAVGSLCALSTSLLGSLFPLPRIIFAMAR 369

medaka_Slc7a1a_isoform_X1|XP_011481652.1 LTLMMPYYLLDSNSPLPVAFKYVGWGGAKYAVAVGSLCALSTSLLGAMFPMPRVIWAMAD 372

medaka_Slc7a1a_isoform_X2|XP_011481655.1 LTLMMPYYLLDSNSPLPVAFKYVGWGGAKYAVAVGSLCALSTSLLGSMFPLPRVIFAMAR 372

fugu_rubripes_Slc7a1a|XP_011618220.1 LTLMMPYYMLDSNSPLPVAFRYVGWEGAKYAVAVGSLCALSTSLLGSMFPLPRIIYAMAR 373

Atlantic_herring_Slc7a1b_isoform_X1|XP_031428872.1 LTMMMPYYLLDKNSPLPLAFTYVGWNGATYAVAVGSLCALSTSLLGSMFPMPRVIWAMAE 370

Atlantic_herring_Slc7a1b_isoform_X2|XP_012686374.2 LTMMMPYYLLDKNSPLPLAFTYVGWNGATYAVAVGSLCALSTSLLGSMFPLPRIIYAMAR 370

zebrafish_Slc7a1b_isoform_X1|NP_001315138.1 LTMMMPYYMLDKNSPLPVAFKYVGWEGATYAVAVGSLCALSTSLLGSMFPMPRVIWAMAE 367

zebrafish_Slc7a1b_isoform_X2|XP_021337047.1 LTMMMPYYMLDKNSPLPVAFKYVGWEGATYAVAVGSLCALSTSLLGSMFPLPRIIFAMAN 367

medaka_Slc7a1b_isoform_X1|XP_011480624.1 LTMMMPYYMLSKDSPLPVAFEYVGWKGATYAVAIGSLCALSTSLLGSMFPMPRVIWAMAE 369

medaka_Slc7a1b_isoform_X2|XP_020563769.1 LTMMMPYYMLSKDSPLPVAFEYVGWKGATYAVAIGSLCALSTSLLGSMFPLPRIIFAMAR 369

fugu_rubripes_Slc7a1b_isoform_X1|XP_029699601.1 LTVMMPYYLLDKNSPLPVAFKYVGWDGATYAVAIGSLCALSTSLLVGMLPMPRVMWAMAK 417

fugu_rubripes_Slc7a1b_isoform_X2|XP_029699602.1 LTVMMPYYLLDKNSPLPVAFKYVGWDGATYAVAIGSLCALSTSLLGSMFPLPRIIFAMAR 417

fugu_rubripes_Slc7a1b_isoform_X3|XP_029699603.1 LTVMMPYYLLDKNSPLPVAFKYVGWDGATYAVAIGSLCALSTSLLVGMLPMPRVMWAMAK 373

**:****: *. :**** ** : ** * ****:*******:*** .::*:**:::***

**<---------9--------> <---------10--------**

human_SLC7A1|NP_003036.1 DGLLFKFLANVNDRTKTPIIATLASGAVAAVMAFLFDLKDLVDLMSIGTLLAYSLVAACV 427

rhesus_monkey_SLC7A1|NP_001253544.1 DGLLFKFLAKVNDRTKTPIIATLASGAIAAVMAFLFDLKDLVDLMSIGTLLAYSLVAACV 427

cattle_SLC7A1|NP_001129264.1 DGLLFKFLAKINDRTKTPIIATLTSGAIAAVMAFLFDLKDLVDLMSIGTLLAYSLVAACV 427

mouse_Slc7a1|NP_001288353.1 DGLLFKFLAKINNRTKTPVIATVTSGAIAAVMAFLFELKDLVDLMSIGTLLAYSLVAACV 420

chicken_SLC7A1|NP_001138962.1 DGLLFKFLAKVNDKRKTPVIATVTSGAVAAIMAFLFDLKDLVDLMSIGTLLAYSLVAACV 428

tropical_clawed_frog_Slc7a1|XP_002934074.1 DGLLFKFLAKVSEKTKTPVIATLTSGSVAALMALLFDLKDLVDLMSIGTLLAYSLVAACV 429

Atlantic_herring_Slc7a1a_isoform_X1|XP_012683156.1 DGLLFKYMADIHPRTKTPLLATLTSGIVAAVMAFLFDLKDLVDLMSIGTLLAYTLVAACV 429

Atlantic_herring_Slc7a1a_isoform_X2|XP_012683159.1 DGLLFSYLARVSER-RTPIMSTMAAGAMSAVMAFLFDLKDLVDLMSIGTLLAYTLVAACV 428

zebrafish_Slc7a1a_isoform_X1|XP_683623.4 DGLLFKFMAGISERTKTPIKATIMSGFLAAIMAFLFDLKDLVDLMSIGTLLAYTLVAACV 429

zebrafish_Slc7a1a_isoform_X2|XP_005155334.1 DGLLFSFLARVSEK-KTPVVSTLASGVTAAIMAFLFDLKDLVDLMSIGTLLAYTLVAACV 428

medaka_Slc7a1a_isoform_X1|XP_011481652.1 DGLLFKFMAEISPRTKTPLIATFASGTGAAIMAFLFDLKDLVDLMSIGTLLAYTLVAACV 432

medaka_Slc7a1a_isoform_X2|XP_011481655.1 DGLLFSFLARISER-KAPVNSTVAAGVMSAIMAFLFDLKDLVDLMSIGTLLAYTLVAACV 431

fugu_rubripes_Slc7a1a|XP_011618220.1 DGLLFSFLARISER-KSPVTSTVTAGVMSAVMAFLFDLKDLVDLMSIGTLLAYTLVAACV 432

Atlantic_herring_Slc7a1b_isoform_X1|XP_031428872.1 DGLLFKFLANISPRTKTPIIATLTSGTVAAIMAFLFDLKALVDLMSIGTLLAYTLVAACV 430

Atlantic_herring_Slc7a1b_isoform_X2|XP_012686374.2 DGLLFSFLARVSER-KTPMVATAASGFMSAIMAFLFDLKALVDLMSIGTLLAYTLVAACV 429

zebrafish_Slc7a1b_isoform_X1|NP_001315138.1 DGLLFKFLANISEKSKTPIMATVTSGIVAAIMAFLFDLKDLVDLMSIGTLLAYTLVAACV 427

zebrafish_Slc7a1b_isoform_X2|XP_021337047.1 DGLIFSFLSRVSER-KTPIVATTGAGLLSAIMAFLFDLKDLVDLMSIGTLLAYTLVAACV 426

medaka_Slc7a1b_isoform_X1|XP_011480624.1 DGLLFKCLASVSSRTKTPLTATVTSGVAAAVMAFLFDLTDLVNLMSIGTLLAYTLVAACV 429

medaka_Slc7a1b_isoform_X2|XP_020563769.1 DGLLFSFLAHVSER-KTPTVSTLVAGLMSAVMAFLFDLTDLVNLMSIGTLLAYTLVAACV 428

fugu_rubripes_Slc7a1b_isoform_X1|XP_029699601.1 DGLLFKSLANISPRTKTPVAATLISGAWAAVMAFLFDLKDLVDLMSIGTLLAYSLVAACV 477

fugu_rubripes_Slc7a1b_isoform_X2|XP_029699602.1 DGLLYSFLARVSER-KTPILSTMVAGLLSAVMAFLFDLKDLVDLMSIGTLLAYSLVAACV 476

fugu_rubripes_Slc7a1b_isoform_X3|XP_029699603.1 DGLLFKSLANISPRTKTPVAATLISGAWAAVMAFLFDLKDLVDLMSIGTLLAYSLVAACV 433

***::. :: : : ::* :* :* :*:**:**:*. **:**********:******

**-->**

human_SLC7A1|NP_003036.1 LVLRYQPEQPNLVY--QMASTSDELDPADQNELASTNDSQLGFLPEA-EMFSLKTILSPK 484

rhesus_monkey_SLC7A1|NP_001253544.1 LVLRYQPEQPNLVY--QMASTSDELDQADQNELASSNDSQLGFLPEA-ELFSLKTILSPK 484

cattle_SLC7A1|NP_001129264.1 LVLRYQPEQPNTVY--QMARTSDELDPVDQNELVSSSDSQTGFLPEA-ERLSLKTILSPK 484

mouse_Slc7a1|NP_001288353.1 LVLRYQPEQPNLVY--QMARTTEELDRVDQNELVSASESQTGFLPVA-EKFSLKSILSPK 477

chicken_SLC7A1|NP_001138962.1 LVLRYQPEQPNLAY--QMARTTEET---DNNESVSTSESQTGFLPEEEEKCSLKAILCPP 483

tropical_clawed_frog_Slc7a1|XP_002934074.1 LVLRYQPDQPNLAY--QMASTNDDPTE--Q--TETSEGSQVGFIVE--DKFTFSLLLFNQ 481

Atlantic_herring_Slc7a1a_isoform_X1|XP_012683156.1 LVLRYQPEHPSMAY--QMASTQEEVEL-----TESMGAPSMVILPGANERFSLKTLLFPE 482

Atlantic_herring_Slc7a1a_isoform_X2|XP_012683159.1 LVLRYQPEHPSMAY--QMASTQEEVEL-----TESMGAPSMVILPGANERFSLKTLLFPE 481

zebrafish_Slc7a1a_isoform_X1|XP_683623.4 LVLRYQPEQFSQTY--HIANTHEDMEM-----SETISTPSMGILPGVEERFSFKNLLFPD 482

zebrafish_Slc7a1a_isoform_X2|XP_005155334.1 LVLRYQPEQFSQTY--HIANTHEDMEM-----SETISTPSMGILPGVEERFSFKNLLFPD 481

medaka_Slc7a1a_isoform_X1|XP_011481652.1 LVLRYQPEHPSHMY--ETVSD----DL-----SDGISVPSMGMLPGVEERFSFHNLLFPD 481

medaka_Slc7a1a_isoform_X2|XP_011481655.1 LVLRYQPEHPSHMY--ETVSD----DL-----SDGISVPSMGMLPGVEERFSFHNLLFPD 480

fugu_rubripes_Slc7a1a|XP_011618220.1 LVLRYQPERPSLVM--ASSPE----EA-----ELSDSNPSMNMLPGLEERFSFKTLLFPD 481

Atlantic_herring_Slc7a1b_isoform_X1|XP_031428872.1 LVLRYQPNQPSCVLPYQAASQEEAEME-----ADSNMESNSGFLPAQ-ERFCMQNVLFPK 484

Atlantic_herring_Slc7a1b_isoform_X2|XP_012686374.2 LVLRYQPNQPSCVLPYQAASQEEAEME-----ADSNMESNSGFLPAQ-ERFCMQNVLFPK 483

zebrafish_Slc7a1b_isoform_X1|NP_001315138.1 LVLRYQPEQPSVNVQYQRASCQE-ETE-----AESINESSAGFLPGSSDLFSLRNLLSPQ 481

zebrafish_Slc7a1b_isoform_X2|XP_021337047.1 LVLRYQPEQPSVNVQYQRASCQE-ETE-----AESINESSAGFLPGSSDLFSLRNLLSPQ 480

medaka_Slc7a1b_isoform_X1|XP_011480624.1 LVLRYQPEQLRAAY--EMAKTQDEADI-----SESY---S-DILPQPEDRLTVKNLLFPS 478

medaka_Slc7a1b_isoform_X2|XP_020563769.1 LVLRYQPEQLRAAY--EMAKTQDEADI-----SESY---S-DILPQPEDRLTVKNLLFPS 477

fugu_rubripes_Slc7a1b_isoform_X1|XP_029699601.1 LILRYRPEHPTSAY--EMANTQEELGT-----TDSY---KEDILPPPEDRFTLRNLFVPS 527

fugu_rubripes_Slc7a1b_isoform_X2|XP_029699602.1 LILRYRPEHPTSAY--EMANTQEELGT-----TDSY---KEDILPPPEDRFTLRNLFVPS 526

fugu_rubripes_Slc7a1b_isoform_X3|XP_029699603.1 LILRYRPEHPTSAY--EMANTQEELGT-----TDSY---KEDILPPPEDRFTLRNLFVPS 483

*:***:*:: . :: : . ::

**<----------11-----------> <------------12----------**

human_SLC7A1|NP_003036.1 NMEPSKISGLIVNISTSLIAVLIITFCIVTVLGREALTKGALWAVFLLAGSALLCAVVTG 544

rhesus_monkey_SLC7A1|NP_001253544.1 NMEPSKISGLIVNISTSLIAVLIITFCIVTVLGREALTKGALWAVFMLAGSALLCAVVTG 544

cattle_SLC7A1|NP_001129264.1 NTEPSKFSGLIVNISTSLLALLVITFCLAAVLGKDALVKGELWAVFLLMGSAFLCSVVTA 544

mouse_Slc7a1|NP_001288353.1 NVEPSKFSGLIVNISAGLLAALIITVCIVAVLGREALAEGTLWAVFVMTGSVLLCMLVTG 537

chicken_SLC7A1|NP_001138962.1 NSDPSKFSGLVVNISTCIMGFLIGGSCVLTTLKPSTLIKA-VWII-----AAILVLIISF 537

tropical_clawed_frog_Slc7a1|XP_002934074.1 NSEPSRSSGSIVNVSAGLIGLLVIVFCCLAVLGQKSMLSGDPSILVPLAVTALLGLFLTI 541

Atlantic_herring_Slc7a1a_isoform_X1|XP_012683156.1 NSDPSPTSGFSVNVCTSLLGLLILTFSILAVQGG-----AAWWNIVALSVLFMVCLGLVF 537

Atlantic_herring_Slc7a1a_isoform_X2|XP_012683159.1 NSDPSPTSGFSVNVCTSLLGLLILTFSILAVQGG-----AAWWNIVALSVLFMVCLGLVF 536

zebrafish_Slc7a1a_isoform_X1|XP_683623.4 IIEPSNLSGFTVNICTSLLGLLILSFSLLAVRGG-----IASWNIITLAVLFGLCVIVTF 537

zebrafish_Slc7a1a_isoform_X2|XP_005155334.1 IIEPSNLSGFTVNICTSLLGLLILSFSLLAVRGG-----IASWNIITLAVLFGLCVIVTF 536

medaka_Slc7a1a_isoform_X1|XP_011481652.1 HPDPSTLSGFTVNICTSALGLLILAFSILAVQAG-----IAVWNMVALSVIFMVCVLLVF 536

medaka_Slc7a1a_isoform_X2|XP_011481655.1 HPDPSTLSGFTVNICTSALGLLILAFSILAVQAG-----IAVWNMVALSVIFMVCVLLVF 535

fugu_rubripes_Slc7a1a|XP_011618220.1 NPEPSKLSGFTVNVCASVLGLLILAFSILAVQGG-----TAVWNIVALTVIFMACLLLGF 536

Atlantic_herring_Slc7a1b_isoform_X1|XP_031428872.1 NTEPSRLSGSVVNIATSVLGLLVCVFCVVAAQETV----LAIWSLLLLVVLAVVCLLITV 540

Atlantic_herring_Slc7a1b_isoform_X2|XP_012686374.2 NTEPSRLSGSVVNIATSVLGLLVCVFCVVAAQETV----LAIWSLLLLVVLAVVCLLITV 539

zebrafish_Slc7a1b_isoform_X1|NP_001315138.1 NEEPSRLSGLTVNICTSILGVLVCVFCVVAVQGG-----FQTWTLAVLISLALVCLIITL 536

zebrafish_Slc7a1b_isoform_X2|XP_021337047.1 NEEPSRLSGLTVNICTSILGVLVCVFCVVAVQGG-----FQTWTLAVLISLALVCLIITL 535

medaka_Slc7a1b_isoform_X1|XP_011480624.1 KPEPSPQSGLVVNICTSILGVLVCVFGVVAVQGG-----LAPWSVCLLSTIAATCLVVTF 533

medaka_Slc7a1b_isoform_X2|XP_020563769.1 KPEPSPQSGLVVNICTSILGVLVCVFGVVAVQGG-----LAPWSVCLLSTIAATCLVVTF 532

fugu_rubripes_Slc7a1b_isoform_X1|XP_029699601.1 CTEPSPQSGSVVSVCTCVLGVLVFVFSVVAVHGG-----FQTWSLSVLGVILALSLMLTF 582

fugu_rubripes_Slc7a1b_isoform_X2|XP_029699602.1 CTEPSPQSGSVVSVCTCVLGVLVFVFSVVAVHGG-----FQTWSLSVLGVILALSLMLTF 581

fugu_rubripes_Slc7a1b_isoform_X3|XP_029699603.1 CTEPSPQSGSVVSVCTCVLGVLVFVFSVVAVHGG-----FQTWSLSVLGVILALSLMLTF 538

:** ** *.:.: :. *: :. : :

**---> <------------13---------> <---------14---------**

human_SLC7A1|NP_003036.1 VIWRQPESKTKLSFKVPFLPVLPILSIFVNVYLMMQLDQGTWVRFAVWMLIGFIIYFGYG 604

rhesus_monkey_SLC7A1|NP_001253544.1 VIWRQPESKTKLSFKVPFLPVLPVLSIFVNVYLMMQLDQGTWVRFAVWMLIGFIIYFGYG 604

cattle_SLC7A1|NP_001129264.1 IIWRQPESKTKLSFKVPFLPVLPVLSIFVNVYLMMQLDKGTWVRFAVWMLIGFFIYFGYG 604

mouse_Slc7a1|NP_001288353.1 IIWRQPESKTKLSFKVPFVPVLPVLSIFVNIYLMMQLDQGTWVRFAVWMLIGFTIYFGYG 597

chicken_SLC7A1|NP_001138962.1 IVWKQPESKTKLSFKVPLLPLLPIVSIFVNVYLMMQLDLGTWIRFAVWMLIGFIIYFSYG 597

tropical_clawed_frog_Slc7a1|XP_002934074.1 VIWRQPESKTKLSFKVPLLPVLPILSILVNVYLMMQLDKGTWIRFTIWMVIGLFIYFGYG 601

Atlantic_herring_Slc7a1a_isoform_X1|XP_012683156.1 IISRQPQSRTKLSFKVPLLPFLPVVSMFVNVYLMMQLDKGTWVRFAIWMAIGFVIYFGYG 597

Atlantic_herring_Slc7a1a_isoform_X2|XP_012683159.1 IISRQPQSRTKLSFKVPLLPFLPVVSMFVNVYLMMQLDKGTWVRFAIWMAIGFVIYFGYG 596

zebrafish_Slc7a1a_isoform_X1|XP_683623.4 IIWRQPESKTKLSFKVPCLPFIPVVSMFVNVYLMMQLDRGTWIRFAIWMSIGLVIYFGYG 597

zebrafish_Slc7a1a_isoform_X2|XP_005155334.1 IIWRQPESKTKLSFKVPCLPFIPVVSMFVNVYLMMQLDRGTWIRFAIWMSIGLVIYFGYG 596

medaka_Slc7a1a_isoform_X1|XP_011481652.1 IVCRQPESRTPLSFKVPLVPFIPVISMFVNVYLMMQLDRGTWVRFSVWMAIGLVIYFCYG 596

medaka_Slc7a1a_isoform_X2|XP_011481655.1 IVCRQPESRTPLSFKVPLVPFIPVISMFVNVYLMMQLDRGTWVRFSVWMAIGLVIYFCYG 595

fugu_rubripes_Slc7a1a|XP_011618220.1 VIWRQPESKTKLSFKVPLLPFIPVISMFVNVYLMMQLDRGTWTRFAIWMVLGFTIYFGYG 596

Atlantic_herring_Slc7a1b_isoform_X1|XP_031428872.1 VIGRQPQSKTKLSFKVPLLPVLPVVSMFINVYLMMQLGRGTWIRFVIWMILGFIIYFGYG 600

Atlantic_herring_Slc7a1b_isoform_X2|XP_012686374.2 VIGRQPQSKTKLSFKVPLLPVLPVVSMFINVYLMMQLGRGTWIRFVIWMILGFIIYFGYG 599

zebrafish_Slc7a1b_isoform_X1|NP_001315138.1 LIWRQPESKTKLSFKVPLLPFLPVLSMFINVYLMMQLDKGTWMRFAIWMVIGFIIYFGYG 596

zebrafish_Slc7a1b_isoform_X2|XP_021337047.1 LIWRQPESKTKLSFKVPLLPFLPVLSMFINVYLMMQLDKGTWMRFAIWMVIGFIIYFGYG 595

medaka_Slc7a1b_isoform_X1|XP_011480624.1 IVWRQPQSKTKLAFKVPLLPFVPVISMFVNVYLMMQLDRGTWIRFAIWMVLGFIIYFTYG 593

medaka_Slc7a1b_isoform_X2|XP_020563769.1 IVWRQPQSKTKLAFKVPLLPFVPVISMFVNVYLMMQLDRGTWIRFAIWMVLGFIIYFTYG 592

fugu_rubripes_Slc7a1b_isoform_X1|XP_029699601.1 VVWRQPQSSAKLVFKVPLLPFLPVASLFINIYLMMQLDKGTWMRFAIWMTLGFLIYFTYG 642

fugu_rubripes_Slc7a1b_isoform_X2|XP_029699602.1 VVWRQPQSSAKLVFKVPLLPFLPVASLFINIYLMMQLDKGTWMRFAIWMTLGFLIYFTYG 641

fugu_rubripes_Slc7a1b_isoform_X3|XP_029699603.1 VVWRQPQSSAKLVFKVPLLPFLPVASLFINIYLMMQLDKGTWMRFAIWMTLGFLIYFTYG 598

:: :**:* : * **** :*.:*: *:::*:******. *** ** :** :*: *** **

**->**

human_SLC7A1|NP_003036.1 LWHSEEASLDAD--QAR----TPDGN--LD---QCK------------------------ 629

rhesus_monkey_SLC7A1|NP_001253544.1 LWHSEEASLDAD--QAR----TPDGN--LD---QCK------------------------ 629

cattle_SLC7A1|NP_001129264.1 LWHSEEATLAAD--PSR----TPDGH--LD---HCK------------------------ 629

mouse_Slc7a1|NP_001288353.1 IWHSEEASLAAG--QAK----TPDSN--LD---QCK------------------------ 622

chicken_SLC7A1|NP_001138962.1 IWHSVEAAYAASADTER----SMDTA--SD---SCK------------------------ 624

tropical_clawed_frog_Slc7a1|XP_002934074.1 MWHSSEAASSETASVPP--NYSPSEDVPAD---ETEKQVMLKGSATEN-SITWEITGED- 654

Atlantic_herring_Slc7a1a_isoform_X1|XP_012683156.1 IRHSAEAGLSRKIPDEELRDYKPISASNGDARASPEKEAFLSNGLSARADDDEGDLLESS 657

Atlantic_herring_Slc7a1a_isoform_X2|XP_012683159.1 IRHSAEAGLSRKIPDEELRDYKPISASNGDARASPEKEAFLSNGLSARADDDEGDLLESS 656

zebrafish_Slc7a1a_isoform_X1|XP_683623.4 IWHSTEAALAHSSMDEELSVYKPACGLNRD-SVTPEKEAFLCNGHGSQVDDDG-DI---- 651

zebrafish_Slc7a1a_isoform_X2|XP_005155334.1 IWHSTEAALAHSSMDEELSVYKPACGLNRD-SVTPEKEAFLCNGHGSQVDDDG-DI---- 650

medaka_Slc7a1a_isoform_X1|XP_011481652.1 IRHSTEGSASHSPLATEMNGLKLE---HELETMATEKEAFLHDGIDVREENGRDL----- 648

medaka_Slc7a1a_isoform_X2|XP_011481655.1 IRHSTEGSASHSPLATEMNGLKLE---HELETMATEKEAFLHDGIDVREENGRDL----- 647

fugu_rubripes_Slc7a1a|XP_011618220.1 IRHSAEAAAARRSSETEMIGFSHE---DKSERVSPEKEAFLHYAIEDREDEDRSL----- 648

Atlantic_herring_Slc7a1b_isoform_X1|XP_031428872.1 IWNSTEAALAKSNATADITSFKDL---MNGHATTPEKTAFLGKGADAQGEEEEEDSDP-- 655

Atlantic_herring_Slc7a1b_isoform_X2|XP_012686374.2 IWNSTEAALAKSNATADITSFKDL---MNGHATTPEKTAFLGKGADAQGEEEEEDSDP-- 654

zebrafish_Slc7a1b_isoform_X1|NP_001315138.1 IWHSTEAALARSE-TEDINAFKPT---TTDEAATPEKEAFLGNCINDRAEEN---SDP-- 647

zebrafish_Slc7a1b_isoform_X2|XP_021337047.1 IWHSTEAALARSE-TEDINAFKPT---TTDEAATPEKEAFLGNCINDRAEEN---SDP-- 646

medaka_Slc7a1b_isoform_X1|XP_011480624.1 IRNSAEAAANRSDADSPACA-------IKGEPMTTEKEAFLHNTQTYSPDEDL------- 639

medaka_Slc7a1b_isoform_X2|XP_020563769.1 IRNSAEAAANRSDADSPACA-------IKGEPMTTEKEAFLHNTQTYSPDEDL------- 638

fugu_rubripes_Slc7a1b_isoform_X1|XP_029699601.1 IRKSAEAVV----TSTPACK-------IKGQPMIVEREAFYHNTTGDDEDS--------- 682

fugu_rubripes_Slc7a1b_isoform_X2|XP_029699602.1 IRKSAEAVV----TSTPACK-------IKGQPMIVEREAFYHNTTGDDEDS--------- 681

fugu_rubripes_Slc7a1b_isoform_X3|XP_029699603.1 IRKSAEAVV----TSTPACK-------IKGQPMIVEREAFYHNTTGDDEDS--------- 638

: :* *. :

**Fig. S1.** Amino acid sequence alignment of teleost fish (Atlantic herring, zebrafish, medaka, fugu rubripes), amphibian (tropical clawed frog), bird (chicken) and mammalian (human, rhesus monkey, cattle, mouse) Slc7a1-type proteins. Multiple sequence alignment was generated using Clustal Omega at <https://www.ebi.ac.uk/Tools/msa/clustalo/> using default parameters. The putative transmembrane domains are named 1 to 14, and are indicated in gray and by double-headed broken arrows (with darker gray in the arrows designating the core stretch of amino acids within each putative predicted transmembrane segment that is shared by all the aligned sequences). Putative N-glycosylation sites are marked in green. Putative protein kinase C phosphorylation sites are also marked in light blue. The E^107^ residue (Wang et al., 1994) is marked in red. Occasionally, marked sites/domains appear superimposed.

1: tropical_clawed_frog_Slc7a1|XP_002934074.1 100.00 71.08 70.34 71.47 71.79 71.63 67.36 66.83 64.74 64.96 64.96 67.40 64.72 64.20 68.02 68.28 66.98 66.30 66.41 65.42 69.34 68.19

2: chicken_SLC7A1|NP_001138962.1 71.08 100.00 76.35 76.61 76.77 76.13 70.53 69.65 67.05 67.11 67.11 69.90 69.24 68.86 71.45 72.06 72.27 71.73 69.61 69.07 72.74 71.38

3: mouse_Slc7a1|NP_001288353.1 70.34 76.35 100.00 87.78 87.14 87.62 68.94 67.55 66.17 67.05 67.05 70.25 68.32 67.77 70.42 69.89 70.92 69.72 69.07 67.87 72.70 71.17

4: cattle_SLC7A1|NP_001129264.1 71.47 76.61 87.78 100.00 91.26 91.57 71.38 70.35 67.49 68.20 68.20 69.61 68.79 67.92 71.64 70.94 72.12 70.94 70.50 68.99 73.78 72.43

5: human_SLC7A1|NP_003036.1 71.79 76.77 87.14 91.26 100.00 98.57 70.07 69.19 66.83 67.38 67.38 69.12 68.14 67.27 70.83 70.62 71.47 70.78 69.85 68.67 73.45 72.27

6: rhesus_monkey_SLC7A1|NP_001253544.1 71.63 76.13 87.62 91.57 98.57 100.00 69.90 69.03 67.00 67.38 67.38 69.12 68.14 67.27 70.83 70.62 71.31 70.78 69.69 68.83 73.62 72.76

7: medaka_Slc7a1b_isoform_X1|XP_011480624.1 67.36 70.53 68.94 71.38 70.07 69.90 100.00 97.34 75.00 75.51 75.51 73.66 71.81 71.14 74.41 74.06 73.82 72.68 72.41 71.43 75.20 73.89

8: medaka_Slc7a1b_isoform_X2|XP_020563769.1 66.83 69.65 67.55 70.35 69.19 69.03 97.34 100.00 76.90 74.53 74.53 74.61 70.66 72.24 73.58 75.16 72.37 74.25 71.27 72.53 74.05 75.32

9: fugu_rubripes_Slc7a1b_isoform_X2|XP_029699602.1 64.74 67.05 66.17 67.49 66.83 67.00 75.00 76.90 100.00 96.62 96.39 71.43 67.46 69.05 69.15 70.25 70.93 73.14 68.09 69.19 71.38 72.81

10: fugu_rubripes_Slc7a1b_isoform_X1|XP_029699601.1 64.96 67.11 67.05 68.20 67.38 67.38 75.51 74.53 96.62 100.00 100.00 69.68 68.46 67.46 69.67 68.83 72.08 70.77 69.09 67.61 71.75 70.43

11: fugu_rubripes_Slc7a1b_isoform_X3|XP_029699603.1 64.96 67.11 67.05 68.20 67.38 67.38 75.51 74.53 96.39 100.00 100.00 69.68 68.46 67.46 69.67 68.83 72.08 70.77 69.09 67.61 71.75 70.43

12: fugu_rubripes_Slc7a1a|XP_011618220.1 67.40 69.90 70.25 69.61 69.12 69.12 73.66 74.61 71.43 69.68 69.68 100.00 79.13 81.30 78.82 80.06 77.17 78.73 71.27 72.36 73.98 74.29

13: medaka_Slc7a1a_isoform_X1|XP_011481652.1 64.72 69.24 68.32 68.79 68.14 68.14 71.81 70.66 67.46 68.46 68.46 79.13 100.00 97.37 79.94 78.50 78.14 76.40 71.32 70.03 73.40 72.26

14: medaka_Slc7a1a_isoform_X2|XP_011481655.1 64.20 68.86 67.77 67.92 67.27 67.27 71.14 72.24 69.05 67.46 67.46 81.30 97.37 100.00 78.50 79.75 76.40 78.11 70.03 70.96 73.04 73.35

15: zebrafish_Slc7a1a_isoform_X1|XP_683623.4 68.02 71.45 70.42 71.64 70.83 70.83 74.41 73.58 69.15 69.67 69.67 78.82 79.94 78.50 100.00 97.08 80.65 79.38 73.11 72.29 78.00 77.03

16: zebrafish_Slc7a1a_isoform_X2|XP_005155334.1 68.28 72.06 69.89 70.94 70.62 70.62 74.06 75.16 70.25 68.83 68.83 80.06 78.50 79.75 97.08 100.00 79.38 80.77 72.29 73.37 77.50 77.81

17: Atlantic_herring_Slc7a1a_isoform_X1|XP_012683156.1 66.98 72.27 70.92 72.12 71.47 71.31 73.82 72.37 70.93 72.08 72.08 77.17 78.14 76.40 80.65 79.38 100.00 96.80 72.20 70.46 76.71 74.96

18: Atlantic_herring_Slc7a1a_isoform_X2|XP_012683159.1 66.30 71.73 69.72 70.94 70.78 70.78 72.68 74.25 73.14 70.77 70.77 78.73 76.40 78.11 79.38 80.77 96.80 100.00 70.62 72.00 75.74 76.67

19: Atlantic_herring_Slc7a1b_isoform_X1|XP_031428872.1 66.41 69.61 69.07 70.50 69.85 69.69 72.41 71.27 68.09 69.09 69.09 71.27 71.32 70.03 73.11 72.29 72.20 70.62 100.00 97.71 79.10 77.36

20: Atlantic_herring_Slc7a1b_isoform_X2|XP_012686374.2 65.42 69.07 67.87 68.99 68.67 68.83 71.43 72.53 69.19 67.61 67.61 72.36 70.03 70.96 72.29 73.37 70.46 72.00 97.71 100.00 77.67 78.45

21: zebrafish_Slc7a1b_isoform_X1|NP_001315138.1 69.34 72.74 72.70 73.78 73.45 73.62 75.20 74.05 71.38 71.75 71.75 73.98 73.40 73.04 78.00 77.50 76.71 75.74 79.10 77.67 100.00 97.37

22: zebrafish_Slc7a1b_isoform_X2|XP_021337047.1 68.19 71.38 71.17 72.43 72.27 72.76 73.89 75.32 72.81 70.43 70.43 74.29 72.26 73.35 77.03 77.81 74.96 76.67 77.36 78.45 97.37 100.00

**Fig. S2.** Percent Identity Matrix (created by Clustal Omega; see alignment in **Fig. S1**).

**Appendix I**

>human_SLC7A1|NP_003036.1

MGCKVLLNIGQQMLRRKVVDCSREETRLSRCLNTFDLVALGVGSTLGAGVYVLAGAVARENAGPAIVISF

LIAALASVLAGLCYGEFGARVPKTGSAYLYSYVTVGELWAFITGWNLILSYIIGTSSVARAWSATFDELI

GRPIGEFSRTHMTLNAPGVLAENPDIFAVIIILILTGLLTLGVKESAMVNKIFTCINVLVLGFIMVSGFV

KGSVKNWQLTEEDFGNTSGRLCLNNDTKEGKPGVGGFMPFGFSGVLSGAATCFYAFVGFDCIATTGEEVK

NPQKAIPVGIVASLLICFIAYFGVSAALTLMMPYFCLDNNSPLPDAFKHVGWEGAKYAVAVGSLCALSAS

LLGSMFPMPRVIYAMAEDGLLFKFLANVNDRTKTPIIATLASGAVAAVMAFLFDLKDLVDLMSIGTLLAY

SLVAACVLVLRYQPEQPNLVYQMASTSDELDPADQNELASTNDSQLGFLPEAEMFSLKTILSPKNMEPSK

ISGLIVNISTSLIAVLIITFCIVTVLGREALTKGALWAVFLLAGSALLCAVVTGVIWRQPESKTKLSFKV

PFLPVLPILSIFVNVYLMMQLDQGTWVRFAVWMLIGFIIYFGYGLWHSEEASLDADQARTPDGNLDQCK

>rhesus_monkey_SLC7A1|NP_001253544.1

MGCKVLLNIGQQMLRRKVVDCSREETRLSRCLNTFDLVALGVGSTLGAGVYVLAGAVARENAGPAIVISF

LIAALASVLAGLCYGEFGARVPKTGSAYLYSYVTVGELWAFITGWNLILSYIIGTSSVARAWSATFDELI

GRPIGEFSRTHMALNAPGVLAENPDIFAVIIILILTGLLTLGVKESAMVNKIFTCVNVLVLGFIMVSGFV

KGSVKNWQLTEEDFGNTSGRLCLNNDTKEGKPGVGGFMPFGFSGVLSGAATCFYAFVGFDCIATTGEEVK

NPQKAIPVGIVASLLICFIAYFGVSAALTLMMPYFCLDNNSPLPDAFKHVGWEGAKYAVAVGSLCALSAS

LLGSMFPMPRVIYAMAEDGLLFKFLAKVNDRTKTPIIATLASGAIAAVMAFLFDLKDLVDLMSIGTLLAY

SLVAACVLVLRYQPEQPNLVYQMASTSDELDQADQNELASSNDSQLGFLPEAELFSLKTILSPKNMEPSK

ISGLIVNISTSLIAVLIITFCIVTVLGREALTKGALWAVFMLAGSALLCAVVTGVIWRQPESKTKLSFKV

PFLPVLPVLSIFVNVYLMMQLDQGTWVRFAVWMLIGFIIYFGYGLWHSEEASLDADQARTPDGNLDQCK

>cattle_SLC7A1|NP_001129264.1

MGCTMLKGVGQQMLRRKVVDCSREESRLSRCLNTFDLVALGVGSTLGAGVYVLAGAVARENAGPAIVISF

LIAALASVLAGLCYGEFGARVPKTGSAYLYSYVTVGELWAFITGWNLILSYIIGTSSVARAWSATFDELI

GKPIGEFSRTHMALHAPGVLAENPDIFAVIIIVILTGLLTLGVKESAMVNKIFTCINVLVLGFIMVSGFV

KGSIKNWQLTEEDFRNTSGHLCLNNDTKEGKPGVGGFMPFGFSGVLSGAATCFYAFVGFDCIATTGEEVK

NPQKAIPVGIVASLLICFIAYFGVSAALTLMMPYFCLDKDSPLPDAFKHVGWEGAKYAVAVGSLCALSTS

LLGSMFPMPRVIYAMAEDGLLFKFLAKINDRTKTPIIATLTSGAIAAVMAFLFDLKDLVDLMSIGTLLAY

SLVAACVLVLRYQPEQPNTVYQMARTSDELDPVDQNELVSSSDSQTGFLPEAERLSLKTILSPKNTEPSK

FSGLIVNISTSLLALLVITFCLAAVLGKDALVKGELWAVFLLMGSAFLCSVVTAIIWRQPESKTKLSFKV

PFLPVLPVLSIFVNVYLMMQLDKGTWVRFAVWMLIGFFIYFGYGLWHSEEATLAADPSRTPDGHLDHCK

>mouse_Slc7a1|NP_001288353.1

MGCKNLLGLGQQMLRRKVVDCSREESRLSRCLNTYDLVALGVGSTLGAGVYVLAGAVARENAGPAIVISF

LIAALASVLAGLCYGEFGARVPKTGSAYLYSYVTVGELWAFITGWNLILSYIIGTSSVARAWSATFDELI

GKPIGEFSRQHMALNAPGVLAQTPDIFAVIIIIILTGLLTLGVKESAMVNKIFTCINVLVLCFIVVSGFV

KGSIKNWQLTEKNFSCNNNDTNVKYGEGGFMPFGFSGVLSGAATCFYAFVGFDCIATTGEEVKNPQKAIP

VGIVASLLICFIAYFGVSAALTLMMPYFCLDIDSPLPGAFKHQGWEEAKYAVAIGSLCALSTSLLGSMFP

MPRVIYAMAEDGLLFKFLAKINNRTKTPVIATVTSGAIAAVMAFLFELKDLVDLMSIGTLLAYSLVAACV

LVLRYQPEQPNLVYQMARTTEELDRVDQNELVSASESQTGFLPVAEKFSLKSILSPKNVEPSKFSGLIVN

ISAGLLAALIITVCIVAVLGREALAEGTLWAVFVMTGSVLLCMLVTGIIWRQPESKTKLSFKVPFVPVLP

VLSIFVNIYLMMQLDQGTWVRFAVWMLIGFTIYFGYGIWHSEEASLAAGQAKTPDSNLDQCK

>chicken_SLC7A1|NP_001138962.1

MECQKFIKFGNQLLRRKNVDCTREDSRLSRCLNTFDLVALGVGSTLGAGVYVLAGAVARVNAGPAIVISF

LIAALASVLAGLCYGEFGARVPKTGSAYLYSYVTVGELWAFITGWNLILSYVIGTSSVARAWSATFDEII

GGHIEDFCKRYMTMNAPGVLAKYPDIFAVVIIIILTGLLTFGVKESALVNKVFTCINILVIGFVVVSGFV

KGSVKNWQLTERDIYNTSPGIHGDNQTQGEKLYGVGGFMPYGLKGVLSGAATCFYAFVGFDCIATTGEEV

KNPQKAIPIGIVASLLICFVAYFGVSAALTLMMPYYQLDTNSPLPNAFKYVGWDGANYAVAVGSLCALST

SLLGSMFPMPRIIYAMAEDGLLFKFLAKVNDKRKTPVIATVTSGAVAAIMAFLFDLKDLVDLMSIGTLLA

YSLVAACVLVLRYQPEQPNLAYQMARTTEETDNNESVSTSESQTGFLPEEEEKCSLKAILCPPNSDPSKF

SGLVVNISTCIMGFLIGGSCVLTTLKPSTLIKAVWIIAAILVLIISFIVWKQPESKTKLSFKVPLLPLLP

IVSIFVNVYLMMQLDLGTWIRFAVWMLIGFIIYFSYGIWHSVEAAYAASADTERSMDTASDSCK

>tropical_clawed_frog_Slc7a1|XP_002934074.1

MNSGDILLTFGQQLFRRKVVGSDSKESRLSRCLNTFDLVALGVGSTLGAGVYVLAGAVARENAGPAIVLC

FLIAALASVLAGLCYGEFGARVPKTGSAYLYSYVTVGEFLAFVTGWNLILSYVIGTSSVARAWSATFDDL

IGKRIEEYCLAHISMNFPGVLARYPDIFSVLIILVLTGLLSFGVKESALVNKVFTCINVLVLGFVIISGF

VKGSVKNWQLSENDFNVTNVTGLPNGTKEQDLNYGAGGFMPFGFAGVLSGAATCFYAFVGFDCIATTGEE

VKNPQKAIPIGIVASLLICFVAYFGVSAALTLMMPYYLLNKESPLPVAFNYVGWDGARYAVAVGSLCALS

TSLLGSMFPMPRVIFAMADDGLLFKFLAKVSEKTKTPVIATLTSGSVAALMALLFDLKDLVDLMSIGTLL

AYSLVAACVLVLRYQPDQPNLAYQMASTNDDPTEQTETSEGSQVGFIVEDKFTFSLLLFNQNSEPSRSSG

SIVNVSAGLIGLLVIVFCCLAVLGQKSMLSGDPSILVPLAVTALLGLFLTIVIWRQPESKTKLSFKVPLL

PVLPILSILVNVYLMMQLDKGTWIRFTIWMVIGLFIYFGYGMWHSSEAASSETASVPPNYSPSEDVPADE

TEKQVMLKGSATENSITWEITGED

>Atlantic_herring_Slc7a1a_isoform_X1|XP_012683156.1

MVLHKLLDFGKQLLRVKVVDCSIEDSRLSRCLNTFDLVALGVGSTLGAGVYVLAGAVARESAGPAIVLSF

LIAALASVMAGLCYAEFGARVPKTGSAYLYSYVTVGELWAFITGWNLILSYVIGTSSVARAWSATFDELI

GKHIEAFCRLNMPMNAPGILAEYPDMFAVLIIITLTGLLSFGVKESAVVNKVFTCINVLVLVFMVVSGLV

KGTLKNWQLVPEEILNGTNSSLNGTDPALVGVDLGVGGFMPYGFTGVLSGAATCFYAFVGFDCIATTGEE

VKNPQRAIPIGIVSSLLICFVAYFGVSAALTMMMPYYMLDKNSPLPVAFKYVGWEGATYAVAVGSLCALS

TSLLGAMFPMPRVIWAMAEDGLLFKYMADIHPRTKTPLLATLTSGIVAAVMAFLFDLKDLVDLMSIGTLL

AYTLVAACVLVLRYQPEHPSMAYQMASTQEEVELTESMGAPSMVILPGANERFSLKTLLFPENSDPSPTS

GFSVNVCTSLLGLLILTFSILAVQGGAAWWNIVALSVLFMVCLGLVFIISRQPQSRTKLSFKVPLLPFLP

VVSMFVNVYLMMQLDKGTWVRFAIWMAIGFVIYFGYGIRHSAEAGLSRKIPDEELRDYKPISASNGDARA

SPEKEAFLSNGLSARADDDEGDLLESS

>Atlantic_herring_Slc7a1a_isoform_X2|XP_012683159.1

MVLHKLLDFGKQLLRVKVVDCSIEDSRLSRCLNTFDLVALGVGSTLGAGVYVLAGAVARESAGPAIVLSF

LIAALASVMAGLCYAEFGARVPKTGSAYLYSYVTVGELWAFITGWNLILSYVIGTSSVARAWSATFDELI

GKHIEAFCRLNMPMNAPGILAEYPDMFAVLIIITLTGLLSFGVKESAVVNKVFTCINVLVLVFMVVSGLV

KGTLKNWQLVPEEILNGTNSSLNGTDPALVGVDLGVGGFMPYGFTGVLSGAATCFYAFVGFDCIATTGEE

VKNPQRAIPIGIVSSLLICFVAYFGVSAALTMMMPYYMLDKNSPLPVAFKYVGWEGATYAVAVGSLCALS

TSLLGSMFPLPRIIFAMARDGLLFSYLARVSERRTPIMSTMAAGAMSAVMAFLFDLKDLVDLMSIGTLLA

YTLVAACVLVLRYQPEHPSMAYQMASTQEEVELTESMGAPSMVILPGANERFSLKTLLFPENSDPSPTSG

FSVNVCTSLLGLLILTFSILAVQGGAAWWNIVALSVLFMVCLGLVFIISRQPQSRTKLSFKVPLLPFLPV

VSMFVNVYLMMQLDKGTWVRFAIWMAIGFVIYFGYGIRHSAEAGLSRKIPDEELRDYKPISASNGDARAS

PEKEAFLSNGLSARADDDEGDLLESS

>zebrafish_Slc7a1a_isoform_X1|XP_683623.4

MVLKKLLRFGKQLLRVKVVNCNSEESRLSRCLNTFDLVALGVGSTLGAGVYVLAGAVARENAGPAIVLSF

LIAALASVLAGLCYAEFGARVPKTGSAYLYSYVTVGELWAFITGWNLILSYVIGTSSVARAWSATFDELI

GKHIEHFCRQYMSMNAPGVLAEYPDMFSVFIILTLTGLLAFGVKESAMVNKVFTCINILVLLFMVVSGLV

KGTLKNWHLDPDEILNATNSTLNATQPLPSEEMLGQGGFMPFGFTGVLSGAATCFYAFVGFDCIATTGEE

VKNPQRAIPIGIVSSLLICFVAYFGVSAALTMMMPYYMLDKNSPLPVAFKYVGWEGATYAVAVGSLCALS

TSLLGAMFPMPRVLWAMADDGLLFKFMAGISERTKTPIKATIMSGFLAAIMAFLFDLKDLVDLMSIGTLL

AYTLVAACVLVLRYQPEQFSQTYHIANTHEDMEMSETISTPSMGILPGVEERFSFKNLLFPDIIEPSNLS

GFTVNICTSLLGLLILSFSLLAVRGGIASWNIITLAVLFGLCVIVTFIIWRQPESKTKLSFKVPCLPFIP

VVSMFVNVYLMMQLDRGTWIRFAIWMSIGLVIYFGYGIWHSTEAALAHSSMDEELSVYKPACGLNRDSVT

PEKEAFLCNGHGSQVDDDGDI

>zebrafish_Slc7a1a_isoform_X2|XP_005155334.1

MVLKKLLRFGKQLLRVKVVNCNSEESRLSRCLNTFDLVALGVGSTLGAGVYVLAGAVARENAGPAIVLSF

LIAALASVLAGLCYAEFGARVPKTGSAYLYSYVTVGELWAFITGWNLILSYVIGTSSVARAWSATFDELI

GKHIEHFCRQYMSMNAPGVLAEYPDMFSVFIILTLTGLLAFGVKESAMVNKVFTCINILVLLFMVVSGLV

KGTLKNWHLDPDEILNATNSTLNATQPLPSEEMLGQGGFMPFGFTGVLSGAATCFYAFVGFDCIATTGEE

VKNPQRAIPIGIVSSLLICFVAYFGVSAALTMMMPYYMLDKNSPLPVAFKYVGWEGATYAVAVGSLCALS

TSLLGSLFPLPRIIFAMARDGLLFSFLARVSEKKTPVVSTLASGVTAAIMAFLFDLKDLVDLMSIGTLLA

YTLVAACVLVLRYQPEQFSQTYHIANTHEDMEMSETISTPSMGILPGVEERFSFKNLLFPDIIEPSNLSG

FTVNICTSLLGLLILSFSLLAVRGGIASWNIITLAVLFGLCVIVTFIIWRQPESKTKLSFKVPCLPFIPV

VSMFVNVYLMMQLDRGTWIRFAIWMSIGLVIYFGYGIWHSTEAALAHSSMDEELSVYKPACGLNRDSVTP

EKEAFLCNGHGSQVDDDGDI

>medaka_Slc7a1a_isoform_X1|XP_011481652.1

MALQALIHFGKQLLRVKVVNGNSEDSRLSRCLDTFDLVALGVGSTLGAGVYVLAGAVARDNSGPAIVLSF

LIAASASVLAGLCYAEFGARVPKTGSAYLYSYVTVGELWAFITGWNLILSYIIGTSSVARAWSATFDELI

GKHIEQFCRTYMSMSAPGVLAEYPDMFAVFIIFTLTGLLAFGVKESAMVNKVFTCINVLVLLFMVVSGLV

KGTIKNWQINPEEILNGTFTSNSSQNQTEVLPTKEILGAGGFMPFGFTGVLSGAATCFYAFVGFDCIATT

GEEVKNPQRAIPIGIVSSLLICFVAYFGVSAALTLMMPYYLLDSNSPLPVAFKYVGWGGAKYAVAVGSLC

ALSTSLLGAMFPMPRVIWAMADDGLLFKFMAEISPRTKTPLIATFASGTGAAIMAFLFDLKDLVDLMSIG

TLLAYTLVAACVLVLRYQPEHPSHMYETVSDDLSDGISVPSMGMLPGVEERFSFHNLLFPDHPDPSTLSG

FTVNICTSALGLLILAFSILAVQAGIAVWNMVALSVIFMVCVLLVFIVCRQPESRTPLSFKVPLVPFIPV

ISMFVNVYLMMQLDRGTWVRFSVWMAIGLVIYFCYGIRHSTEGSASHSPLATEMNGLKLEHELETMATEK

EAFLHDGIDVREENGRDL

>medaka_Slc7a1a_isoform_X2|XP_011481655.1

MALQALIHFGKQLLRVKVVNGNSEDSRLSRCLDTFDLVALGVGSTLGAGVYVLAGAVARDNSGPAIVLSF

LIAASASVLAGLCYAEFGARVPKTGSAYLYSYVTVGELWAFITGWNLILSYIIGTSSVARAWSATFDELI

GKHIEQFCRTYMSMSAPGVLAEYPDMFAVFIIFTLTGLLAFGVKESAMVNKVFTCINVLVLLFMVVSGLV

KGTIKNWQINPEEILNGTFTSNSSQNQTEVLPTKEILGAGGFMPFGFTGVLSGAATCFYAFVGFDCIATT

GEEVKNPQRAIPIGIVSSLLICFVAYFGVSAALTLMMPYYLLDSNSPLPVAFKYVGWGGAKYAVAVGSLC

ALSTSLLGSMFPLPRVIFAMARDGLLFSFLARISERKAPVNSTVAAGVMSAIMAFLFDLKDLVDLMSIGT

LLAYTLVAACVLVLRYQPEHPSHMYETVSDDLSDGISVPSMGMLPGVEERFSFHNLLFPDHPDPSTLSGF

TVNICTSALGLLILAFSILAVQAGIAVWNMVALSVIFMVCVLLVFIVCRQPESRTPLSFKVPLVPFIPVI

SMFVNVYLMMQLDRGTWVRFSVWMAIGLVIYFCYGIRHSTEGSASHSPLATEMNGLKLEHELETMATEKE

AFLHDGIDVREENGRDL

>fugu_rubripes_Slc7a1a|XP_011618220.1

MALKRLLGFGKQLLRVKVVDCNSEESHLSRCLNTFDLVALGVGSTLGAGVYVLAGAVARENSGPAIVLCF

LIAALASVLAGLCYAEFGARVPKTGSAYLYSYVTVGEIWAFFTGWNLILSYVIGTSSVARAWSATFDELI

GKHIEHFCRAYMSMNAPGVLAEYPDAFAVVIIITLTGLLAFGVKESAMVNKVFTCINVLVLLFMVISGLV

KGTMKNWQIDPEEILKANHTTSNSSLNLTELLPSRESMGAGGFMPFGWSGVLSGAATCFYAFVGFDCIAT

TGEEVKNPQRAIPIGIVSSLLICFVAYFGVSAALTLMMPYYMLDSNSPLPVAFRYVGWEGAKYAVAVGSL

CALSTSLLGSMFPLPRIIYAMARDGLLFSFLARISERKSPVTSTVTAGVMSAVMAFLFDLKDLVDLMSIG

TLLAYTLVAACVLVLRYQPERPSLVMASSPEEAELSDSNPSMNMLPGLEERFSFKTLLFPDNPEPSKLSG

FTVNVCASVLGLLILAFSILAVQGGTAVWNIVALTVIFMACLLLGFVIWRQPESKTKLSFKVPLLPFIPV

ISMFVNVYLMMQLDRGTWTRFAIWMVLGFTIYFGYGIRHSAEAAAARRSSETEMIGFSHEDKSERVSPEK

EAFLHYAIEDREDEDRSL

>Atlantic_herring_Slc7a1b_isoform_X1|XP_031428872.1

MFLSTLKDLGRQLLRVKVVDCSATESRLARCLNTFDLVALGVGSTLGAGVYVLAGAVARENSGPAIVLSF

LIAALASVMAGLCYAEFGARVPKTGSAYLYSYVTVGELWAFITGWNLILSYIIGTSSVARAWSATFDGLI

GNHIEKFCKQYMNMKAEGILAEYPDMFAVLIIITLTGLLAFGVKESAMVNKVFTCVNVLVLLFVVVSGLV

KGTLKNWNIDPEEVLHYNGSGTYNLSETLPTPAEVGAGGFMPFGVKGVLSGAATCFYAFVGFDCIATTGE

EVKNPQRAIPIGIVASLLICFLAYFGVSAALTMMMPYYLLDKNSPLPLAFTYVGWNGATYAVAVGSLCAL

STSLLGSMFPMPRVIWAMAEDGLLFKFLANISPRTKTPIIATLTSGTVAAIMAFLFDLKALVDLMSIGTL

LAYTLVAACVLVLRYQPNQPSCVLPYQAASQEEAEMEADSNMESNSGFLPAQERFCMQNVLFPKNTEPSR

LSGSVVNIATSVLGLLVCVFCVVAAQETVLAIWSLLLLVVLAVVCLLITVVIGRQPQSKTKLSFKVPLLP

VLPVVSMFINVYLMMQLGRGTWIRFVIWMILGFIIYFGYGIWNSTEAALAKSNATADITSFKDLMNGHAT

TPEKTAFLGKGADAQGEEEEEDSDP

>Atlantic_herring_Slc7a1b_isoform_X2|XP_012686374.2

MFLSTLKDLGRQLLRVKVVDCSATESRLARCLNTFDLVALGVGSTLGAGVYVLAGAVARENSGPAIVLSF

LIAALASVMAGLCYAEFGARVPKTGSAYLYSYVTVGELWAFITGWNLILSYIIGTSSVARAWSATFDGLI

GNHIEKFCKQYMNMKAEGILAEYPDMFAVLIIITLTGLLAFGVKESAMVNKVFTCVNVLVLLFVVVSGLV

KGTLKNWNIDPEEVLHYNGSGTYNLSETLPTPAEVGAGGFMPFGVKGVLSGAATCFYAFVGFDCIATTGE

EVKNPQRAIPIGIVASLLICFLAYFGVSAALTMMMPYYLLDKNSPLPLAFTYVGWNGATYAVAVGSLCAL

STSLLGSMFPLPRIIYAMARDGLLFSFLARVSERKTPMVATAASGFMSAIMAFLFDLKALVDLMSIGTLL

AYTLVAACVLVLRYQPNQPSCVLPYQAASQEEAEMEADSNMESNSGFLPAQERFCMQNVLFPKNTEPSRL

SGSVVNIATSVLGLLVCVFCVVAAQETVLAIWSLLLLVVLAVVCLLITVVIGRQPQSKTKLSFKVPLLPV

LPVVSMFINVYLMMQLGRGTWIRFVIWMILGFIIYFGYGIWNSTEAALAKSNATADITSFKDLMNGHATT

PEKTAFLGKGADAQGEEEEEDSDP

>zebrafish_Slc7a1b_isoform_X1|NP_001315138.1

MVLTIMKELGKQLLRVKVVDCSTEESRLSRCLNTFDLVALGVGSTLGAGVYVLAGAVARENAGPAIVLSF

LIAALASVMAGLCYAEFGARVPRTGSAYLYSYVTVGELWAFITGWNLILSYIIGTSSVARAWSATFDELI

GKHIEEFCRRHMSINTPGLAEYPDMFAVIIILTLTGLLAFGVKESAMVNKVFTCVNVLVLMFVVVSGLIK

GTLQNWNLNPEDILNHTTSANVSVPLPTEESLGFGGFMPFGFSGVLSGAATCFYAFVGFDCIATTGEEVK

NPQRAIPIGIVASLLICFVAYFGVSAALTMMMPYYMLDKNSPLPVAFKYVGWEGATYAVAVGSLCALSTS

LLGSMFPMPRVIWAMAEDGLLFKFLANISEKSKTPIMATVTSGIVAAIMAFLFDLKDLVDLMSIGTLLAY

TLVAACVLVLRYQPEQPSVNVQYQRASCQEETEAESINESSAGFLPGSSDLFSLRNLLSPQNEEPSRLSG

LTVNICTSILGVLVCVFCVVAVQGGFQTWTLAVLISLALVCLIITLLIWRQPESKTKLSFKVPLLPFLPV

LSMFINVYLMMQLDKGTWMRFAIWMVIGFIIYFGYGIWHSTEAALARSETEDINAFKPTTTDEAATPEKE

AFLGNCINDRAEENSDP

>zebrafish_Slc7a1b_isoform_X2|XP_021337047.1

MVLTIMKELGKQLLRVKVVDCSTEESRLSRCLNTFDLVALGVGSTLGAGVYVLAGAVARENAGPAIVLSF

LIAALASVMAGLCYAEFGARVPRTGSAYLYSYVTVGELWAFITGWNLILSYIIGTSSVARAWSATFDELI

GKHIEEFCRRHMSINTPGLAEYPDMFAVIIILTLTGLLAFGVKESAMVNKVFTCVNVLVLMFVVVSGLIK

GTLQNWNLNPEDILNHTTSANVSVPLPTEESLGFGGFMPFGFSGVLSGAATCFYAFVGFDCIATTGEEVK

NPQRAIPIGIVASLLICFVAYFGVSAALTMMMPYYMLDKNSPLPVAFKYVGWEGATYAVAVGSLCALSTS

LLGSMFPLPRIIFAMANDGLIFSFLSRVSERKTPIVATTGAGLLSAIMAFLFDLKDLVDLMSIGTLLAYT

LVAACVLVLRYQPEQPSVNVQYQRASCQEETEAESINESSAGFLPGSSDLFSLRNLLSPQNEEPSRLSGL

TVNICTSILGVLVCVFCVVAVQGGFQTWTLAVLISLALVCLIITLLIWRQPESKTKLSFKVPLLPFLPVL

SMFINVYLMMQLDKGTWMRFAIWMVIGFIIYFGYGIWHSTEAALARSETEDINAFKPTTTDEAATPEKEA

FLGNCINDRAEENSDP

>medaka_Slc7a1b_isoform_X1|XP_011480624.1

MLLATLKGLGRKLLRVKVVDCSTEESRLSRCLGTFDLVALGVGSTLGAGVYVLAGAVARENSGPAIVLSF

LIAALASVLAGLCYAEFGARVPRTGSAYLYSYVTVGELWAFITGWNLILSYVIGTSSVARAWSATFDELI

NGHIKRFSSQYLPMNAPGVAEYPDIFAVLIILSLTGLLVFGVKESALVNKVFTCINVLVLLFVIIAGFVK

GDRKNWSLNPEDILNSTRNSTLNTTASLASEETIGAGGFMPFGWTGVLSGAATCFYAFVGFDCIATTGEE

VKNPQRAIPIGIVASLLICFVAYFGVSAALTMMMPYYMLSKDSPLPVAFEYVGWKGATYAVAIGSLCALS

TSLLGSMFPMPRVIWAMAEDGLLFKCLASVSSRTKTPLTATVTSGVAAAVMAFLFDLTDLVNLMSIGTLL

AYTLVAACVLVLRYQPEQLRAAYEMAKTQDEADISESYSDILPQPEDRLTVKNLLFPSKPEPSPQSGLVV

NICTSILGVLVCVFGVVAVQGGLAPWSVCLLSTIAATCLVVTFIVWRQPQSKTKLAFKVPLLPFVPVISM

FVNVYLMMQLDRGTWIRFAIWMVLGFIIYFTYGIRNSAEAAANRSDADSPACAIKGEPMTTEKEAFLHNT

QTYSPDEDL

>medaka_Slc7a1b_isoform_X2|XP_020563769.1

MLLATLKGLGRKLLRVKVVDCSTEESRLSRCLGTFDLVALGVGSTLGAGVYVLAGAVARENSGPAIVLSF

LIAALASVLAGLCYAEFGARVPRTGSAYLYSYVTVGELWAFITGWNLILSYVIGTSSVARAWSATFDELI

NGHIKRFSSQYLPMNAPGVAEYPDIFAVLIILSLTGLLVFGVKESALVNKVFTCINVLVLLFVIIAGFVK

GDRKNWSLNPEDILNSTRNSTLNTTASLASEETIGAGGFMPFGWTGVLSGAATCFYAFVGFDCIATTGEE

VKNPQRAIPIGIVASLLICFVAYFGVSAALTMMMPYYMLSKDSPLPVAFEYVGWKGATYAVAIGSLCALS

TSLLGSMFPLPRIIFAMARDGLLFSFLAHVSERKTPTVSTLVAGLMSAVMAFLFDLTDLVNLMSIGTLLA

YTLVAACVLVLRYQPEQLRAAYEMAKTQDEADISESYSDILPQPEDRLTVKNLLFPSKPEPSPQSGLVVN

ICTSILGVLVCVFGVVAVQGGLAPWSVCLLSTIAATCLVVTFIVWRQPQSKTKLAFKVPLLPFVPVISMF

VNVYLMMQLDRGTWIRFAIWMVLGFIIYFTYGIRNSAEAAANRSDADSPACAIKGEPMTTEKEAFLHNTQ

TYSPDEDL

>fugu_rubripes_Slc7a1b_isoform_X1|XP_029699601.1

MNQSGNCSRDTTAAGKVGFKPEKRDRSLWCPRFLAVVFLLSGAKMRIKMLATLKDIGSRLLRVKVMDCST

EESHFSRCLNTFDLVALGVGSTLGAGVYVLAGAVARDTSGPAIVLSFFIAALASVLAGLCYAEFGARVPK

TGSAYLYTYVTVGELWAFITGWNLILSYVIGTASVARAWSATFDKMIGKYIEEFCRQHMTMDAPGVLAEY

PDIFAVFIIIILTGLLAFGVKESAWVNKVFTSVNVVVLVFVIISGFVKGNLKNWSLNPEEIFNSTSNSSL

NLSSPALSEDVLGAGGFMPFGWSGVLSGAATCFYAFIGFDCIATTGEEVKNPQRAIPVGIVASLLICFVA

YFGVSAALTVMMPYYLLDKNSPLPVAFKYVGWDGATYAVAIGSLCALSTSLLVGMLPMPRVMWAMAKDGL

LFKSLANISPRTKTPVAATLISGAWAAVMAFLFDLKDLVDLMSIGTLLAYSLVAACVLILRYRPEHPTSA

YEMANTQEELGTTDSYKEDILPPPEDRFTLRNLFVPSCTEPSPQSGSVVSVCTCVLGVLVFVFSVVAVHG

GFQTWSLSVLGVILALSLMLTFVVWRQPQSSAKLVFKVPLLPFLPVASLFINIYLMMQLDKGTWMRFAIW

MTLGFLIYFTYGIRKSAEAVVTSTPACKIKGQPMIVEREAFYHNTTGDDEDS

>fugu_rubripes_Slc7a1b_isoform_X2|XP_029699602.1

MNQSGNCSRDTTAAGKVGFKPEKRDRSLWCPRFLAVVFLLSGAKMRIKMLATLKDIGSRLLRVKVMDCST

EESHFSRCLNTFDLVALGVGSTLGAGVYVLAGAVARDTSGPAIVLSFFIAALASVLAGLCYAEFGARVPK

TGSAYLYTYVTVGELWAFITGWNLILSYVIGTASVARAWSATFDKMIGKYIEEFCRQHMTMDAPGVLAEY

PDIFAVFIIIILTGLLAFGVKESAWVNKVFTSVNVVVLVFVIISGFVKGNLKNWSLNPEEIFNSTSNSSL

NLSSPALSEDVLGAGGFMPFGWSGVLSGAATCFYAFIGFDCIATTGEEVKNPQRAIPVGIVASLLICFVA

YFGVSAALTVMMPYYLLDKNSPLPVAFKYVGWDGATYAVAIGSLCALSTSLLGSMFPLPRIIFAMARDGL

LYSFLARVSERKTPILSTMVAGLLSAVMAFLFDLKDLVDLMSIGTLLAYSLVAACVLILRYRPEHPTSAY

EMANTQEELGTTDSYKEDILPPPEDRFTLRNLFVPSCTEPSPQSGSVVSVCTCVLGVLVFVFSVVAVHGG

FQTWSLSVLGVILALSLMLTFVVWRQPQSSAKLVFKVPLLPFLPVASLFINIYLMMQLDKGTWMRFAIWM

TLGFLIYFTYGIRKSAEAVVTSTPACKIKGQPMIVEREAFYHNTTGDDEDS

>fugu_rubripes_Slc7a1b_isoform_X3|XP_029699603.1

MRIKMLATLKDIGSRLLRVKVMDCSTEESHFSRCLNTFDLVALGVGSTLGAGVYVLAGAVARDTSGPAIV

LSFFIAALASVLAGLCYAEFGARVPKTGSAYLYTYVTVGELWAFITGWNLILSYVIGTASVARAWSATFD

KMIGKYIEEFCRQHMTMDAPGVLAEYPDIFAVFIIIILTGLLAFGVKESAWVNKVFTSVNVVVLVFVIIS

GFVKGNLKNWSLNPEEIFNSTSNSSLNLSSPALSEDVLGAGGFMPFGWSGVLSGAATCFYAFIGFDCIAT

TGEEVKNPQRAIPVGIVASLLICFVAYFGVSAALTVMMPYYLLDKNSPLPVAFKYVGWDGATYAVAIGSL

CALSTSLLVGMLPMPRVMWAMAKDGLLFKSLANISPRTKTPVAATLISGAWAAVMAFLFDLKDLVDLMSI

GTLLAYSLVAACVLILRYRPEHPTSAYEMANTQEELGTTDSYKEDILPPPEDRFTLRNLFVPSCTEPSPQ

SGSVVSVCTCVLGVLVFVFSVVAVHGGFQTWSLSVLGVILALSLMLTFVVWRQPQSSAKLVFKVPLLPFL

PVASLFINIYLMMQLDKGTWMRFAIWMTLGFLIYFTYGIRKSAEAVVTSTPACKIKGQPMIVEREAFYHN

TTGDDEDS

>human_CAT-2A|AAB62810.1

MIPCRAALTFARCLIRRKIVTLDSLEDTKLCRCLSTMDLIALGVGSTLGAGVYVLAGEVAKADSGPSIVV

SFLIAALASVMAGLCYAEFGARVPKTGSAYLYTYVTVGELWAFITGWNLILSYVIGTSSVARAWSGTFDE

LLSKQIGQFLRTYFRMNYTGLAEYPDFFAVCLILLLAGLLSFGVKESAWVNKVFTAVNILVLLFVMVAGF

VKGNVANWKISEEFLKNISASAREPPSENGTSIYGAGGFMPYGFTGTLAGAATCFYAFVGFDCIATTGEE

VRNPQKAIPIGIVTSLLVCFMAYFGVSAALTLMMPYYLLDEKSPLPVAFEYVGWGPAKYVVAAGSLCALS

TSLLGSMFPLPRILFAMARDGLLFRFLARVSKRQSPVAATLTAGVISALMAFLFDLKALVDMMSIGTLMA

YSLVAACVLILRYQPGLSYDQPKCSPEKDGLGSSPRVTSKSESQVTMLQRQGFSMRTLFCPSLLPTQQSA

SLVSFLVGFLAFLVLGLSVLTTYGVHAITRLEAWSLALLTLFLVLFVAIVLTIWRQPQNQQKVAFMVPFL

PFLPAFSILVNIYLMVQLSADTWVRFSIWMAIGFLIYFSYGIRHSLEGHLRDENNEEDAYPDNVHAAAEE

KSAIQANDHHPRNLSSPFIFHEKTSEF

>human_CAT-2B|AAI43584.1

MIPCRAALTFARCLIRRKIVTLDSLEDTKLCRCLSTMDLIALGVGSTLGAGVYVLAGEVAKADSGPSIVV

SFLIAALASVMAGLCYAEFGARVPKTGSAYLYTYVTVGELWAFITGWNLILSYVIGTSSVARAWSGTFDE

LLSKQIGQFLRTYFRMNYTGLAEYPDFFAVCLILLLAGLLSFGVKESAWVNKVFTAVNILVLLFVMVAGF

VKGNVANWKISEEFLKNISASAREPPSENGTSIYGAGGFMPYGFTGTLAGAATCFYAFVGFDCIATTGEE

VRNPQKAIPIGIVTSLLVCFMAYFGVSAALTLMMPYYLLDEKSPLPVAFEYVGWGPAKYVVAAGSLCALS

TSLLGSIFPMPRVIYAMAEDGLLFKCLAQINSKTKTPIIATLSSGAVAALMAFLFDLKALVDMMSIGTLM

AYSLVAACVLILRYQPGLSYDQPKCSPEKDGLGSSPRVTSKSESQVTMLQRQGFSMRTLFCPSLLPTQQS

ASLVSFLVGFLAFLVLGLSVLTTYGVHAITRLEAWSLALLALFLVLFVAIVLTIWRQPQNQQKVAFMVPF

LPFLPAFSILVNIYLMVQLSADTWVRFSIWMAIGFLIYFSYGIRHSLEGHLRDENNEEDAYPDNVHAAAE

EKSAIQANDHHPRNLSSPFIFHEKTSEF

>mouse_Cat-2a|XP_006509314.1

MGTESEGQETSQEQPGTHRSISPFTCVGEKANVPRIVCGQVRPQHSRGEQQNSRLQGLLSSTMIPCRAVL

TFARCLIRRKIVTLDSLEDSKLCRCLTTVDLIALGVGSTLGAGVYVLAGEVAKADSGPSIVVSFLIAALA

SVMAGLCYAEFGARVPKTGSAYLYTYVTVGELWAFITGWNLILSYVIGTSSVARAWSGTFDELLNKQIGQ

FFKTYFKMNYTGLAEYPDFFAVCLVLLLAGLLSFGVKESAWVNKFFTAINILVLLFVMVAGFVKGNVANW

KISEEFLKNISASAREPPSENGTSIYGAGGFMPYGFTGTLAGAATCFYAFVGFDCIATTGEEVRNPQKAI

PIGIVTSLLVCFMAYFGVSAALTLMMPYYLLDEKSPLPVAFEYVRWSPAKYVVSAGSLCALSTSLLGSMF

PLPRILFAMARDGLLFRFLARVSKRQSPVAATMTAGVISAVMAFLFDLKALVDMMSIGTLMAYSLVAACV

LILRYQPGLCYDQPKYTPEKETLESCTNATLKSESQVTMLQGQGFSLRTLFSPSALPTRQSASLVSFLVG

FLAFLILGLSILTTYGVQAIARLEAWSLALLALFLVLCVAVILTIWRQPQNQQKVAFMVPFLPFLPAFSI

LVNIYLMVQLSADTWIRFSIWMALGFLIYFAYGIRHSLEGNPRDEEDDEDAFSDNINAATEEKSAMQAND

HHQRNLSLPFILHEKTSEC

>mouse_Cat-2b|XP_006509313.1

MGTESEGQETSQEQPGTHRSISPFTCVGEKANVPRIVCGQVRPQHSRGEQQNSRLQGLLSSTMIPCRAVL

TFARCLIRRKIVTLDSLEDSKLCRCLTTVDLIALGVGSTLGAGVYVLAGEVAKADSGPSIVVSFLIAALA

SVMAGLCYAEFGARVPKTGSAYLYTYVTVGELWAFITGWNLILSYVIGTSSVARAWSGTFDELLNKQIGQ

FFKTYFKMNYTGLAEYPDFFAVCLVLLLAGLLSFGVKESAWVNKFFTAINILVLLFVMVAGFVKGNVANW

KISEEFLKNISASAREPPSENGTSIYGAGGFMPYGFTGTLAGAATCFYAFVGFDCIATTGEEVRNPQKAI

PIGIVTSLLVCFMAYFGVSAALTLMMPYYLLDEKSPLPVAFEYVRWSPAKYVVSAGSLCALSTSLLGSIF

PMPRVIYAMAEDGLLFKCLAQINSKTKTPVIATLSSGAVAAVMAFLFDLKALVDMMSIGTLMAYSLVAAC

VLILRYQPGLCYDQPKYTPEKETLESCTNATLKSESQVTMLQGQGFSLRTLFSPSALPTRQSASLVSFLV

GFLAFLILGLSILTTYGVQAIARLEAWSLALLALFLVLCVAVILTIWRQPQNQQKVAFMVPFLPFLPAFS

ILVNIYLMVQLSADTWIRFSIWMALGFLIYFAYGIRHSLEGNPRDEEDDEDAFSDNINAATEEKSAMQAN

DHHQRNLSLPFILHEKTSEC

>human_SLC7A3|NP_001041629.1

MPWQAFRRFGQKLVRRRTLESGMAETRLARCLSTLDLVALGVGSTLGAGVYVLAGEVAKDKAGPSIVICF

LVAALSSVLAGLCYAEFGARVPRSGSAYLYSYVTVGELWAFTTGWNLILSYVIGTASVARAWSSAFDNLI

GNHISKTLQGSIALHVPHVLAEYPDFFALGLVLLLTGLLALGASESALVTKVFTGVNLLVLGFVMISGFV

KGDVHNWKLTEEDYELAMAELNDTYSLGPLGSGGFVPFGFEGILRGAATCFYAFVGFDCIATTGEEAQNP

QRSIPMGIVISLSVCFLAYFAVSSALTLMMPYYQLQPESPLPEAFLYIGWAPARYVVAVGSLCALSTSLL

GSMFPMPRVIYAMAEDGLLFRVLARIHTGTRTPIIATVVSGIIAAFMAFLFKLTDLVDLMSIGTLLAYSL

VSICVLILRYQPDQETKTGEEVELQEEAITTESEKLTLWGLFFPLNSIPTPLSGQIVYVCSSLLAVLLTA

LCLVLAQWSVPLLSGDLLWTAVVVLLLLLIIGIIVVIWRQPQSSTPLHFKVPALPLLPLMSIFVNIYLMM

QMTAGTWARFGVWMLIGFAIYFGYGIQHSLEEIKSNQPSRKSRAKTVDLDPGTLYVHSV

>mouse_Slc7a3|NP_001288769.1

MLWQALRRFGQKLVRRRVLELGMGETRLARCLSTLDLVALGVGSTLGAGVYVLAGEVAKDKAGPSIVICF

LVAALSSVLAGLCYAEFGARVPGSGSAYLYSYVTVGELWAFTTGWNLILSYVIGTASVARAWSSAFDNLI

GNHISRTLKGTILLKMPHVLAEYPDFFALALVLLLTGLLVLGASKSALVTKVFTGMNLLVLSFVIISGFI

KGELRNWKLTKEDYCLTMSESNGTCSLDSMGSGGFMPFGLEGILRGAATCFYAFVGFDCIATTGEEAQNP

QRSIPMGIVISMFICFLAYFGVSSALTLMMPYYKLHPESPLPEAFSYVGWEPARYLVAIGSLCALSTSLL

GSMFPMPRVMYSMAEDGLLFRVLAKVHSVTHIPIVATLVSGVIAAFMAFLFELTDLVDLMSIGTLLAHSL

VSICVLILRYQPDQEMKSVEEEMELQEETLEAEKLTVQALFCPVNSIPTLLSGRVVYVCSSLLAVLLTVL

CLVLTWWTTPLRSGDPVWVTVVVLILGLILAISGVIWRQPQNRTPLHFKVPAVPLLPLVSIFVNVYLMMQ

MTAGTWARFGIWMLIGFAIYFGYGIQHSMKEVKNHQTLPKTRAQTIDLDLTTSCVHSI

>GkApcT|pdb|5OQT|A Chain A, Amino acid transporter

MNLFRKKPIQLLMKESGAKGASLRKELGAFDLTMLGIGAIIGTGIFVLTGVAAAEHAGPALVLSFILSGL

ACVFAALCYAEFASTVPVSGSAYTYSYATFGELIAWILGWDLILEYGVASSAVAVGWSGYFQGLLSGFGI

ELPKALTSAYDPAKGTFIDLPAIIIVLFITFLLNLGAKKSARFNAVIVAIKVAVVLLFLAVGVWYVKPEN

WTPFMPYGFSGVATGAATVFFAYIGFDAVSTAAEEVRNPQRDMPIGIIVSLLVCTLLYIAVSLVLTGIVP

YEQLNVKNPVAFALNYIHQDWVAGFISLGAIAGITTVLLVMMYGQTRLFYAISRDGLLPKVFARISPTRQ

VPYVNTWLTGAAVAVFAGIIPLNKLAELTNIGTLFAFITVSIGVLVLRKTQPDLKRAFRVPFVPVVPILA

VLFCGYLVLQLPAMTWIGFVSWLLIGLVIYFIYGRKHSELNEMARTEEKAG

(for structural analysis in Fig. 4C only)

>zebrafish_CAT-2A|F1Q9M9

MLEHCLTFGRSLVRRKNVDQGCLEESKLCRCLSTVDLIALGVGSTLGAGVYVLAGEVAKGSSGPSIVVSF

LIAALASVMAGLCYAEFGARVPKTGSAYLYSYVTVGELWAFITGWNLILSYVIGTSSVARAWSGTFDEII

GGHIEKFCKMYFKMSLPGLAEYPDFFAVCLILLLSGLLSFGVKESAWVNKIFTAVNVLVLMFVIISGFVK

GDSLNWNISEESLVNYTVVKRNISSAANVTSDYGAGGFFPYGFGGTLAGAATCFYAFVGFDCIATTGEEV

KNPQRAIPIGIVVSLLVCFLAYFGVSAALTLMMPYYLLDEKSPLPLAFEYVGWGPAKYVVAAGSLCALST

SLLGSMFPLPRILFAMARDGVLFRFLSKLSKRQSPVAATMAAGTTAAIMAFLFDLKALVDMMSIGTLLAY

SLVAACVLILRYQPDASFERSRISEGKEEVGESELTESESHLNMLKDGGVTLRSLLHPPLLPTKNTSSVV

NVSVIITVLVVCVVSTLNTYYGQAIIAMELWALGVLAASLFIFIICVFLICRQPQTRKKVSFMVPLLPFL

PILSIFVNVYLMVQLSGDTWIRFSIWMAIGFLIYFGYGMWHSDERKRHLQNCAVATEKKTLTGNEGFVEN

KTHTEKTSPC

>zebrafish_CAT-2B|A0A286Y7Z6

MLEHCLTFGRSLVRRKNVDQGCLEESKLCRCLSTVDLIALGVGSTLGAGVYVLAGEVAKGSSGPSIVVSF

LIAALASVMAGLCYAEFGARVPKTGSAYLYSYVTVGELWAFITGWNLILSYVIGTSSVARAWSGTFDEII

GGHIEKFCKMYFKMSLPGLAEYPDFFAVCLILLLSGLLSFGVKESAWVNKIFTAVNVLVLMFVIISGFVK

GDSLNWNISEESLVNYTVVKRNISSAANVTSDYGAGGFFPYGFGGTLAGAATCFYAFVGFDCIATTGEEV

KNPQRAIPIGIVVSLLVCFLAYFGVSAALTLMMPYYLLDEKSPLPLAFEYVGWGPAKYVVAAGSLCALST

SLLGSIFPMPRVIYAMAQDGVLFKVLAQINPKTKTPLIATMSSGVVAAIMAFLFDLKALVDMMSIGTLLA

YSLVAACVLILRYQPDASFERSRISEGKEEVGESELTESESHLNMLKDGGVTLRSLLHPPLLPTKNTSSV

VNVSVIITVLVVCVVSTLNTYYGQAIIAMELWALGVLAASLFIFIICVFLICRQPQTRKKVSFMVPLLPF

LPILSIFVNVYLMVQLSGDTWIRFSIWMAIGFLIYFGYGMWHSDERKRHLQNCAVATEKKTLTGNEGFVE

NKTHTEKTSPC

>human_SLC7A4|O43246

MARGLPTIASLARLCQKLNRLKPLEDSTMETSLRRCLSTLDLTLLGVGGMVGSGLYVLTGAVAKEVAGPA

VLLSFGVAAVASLLAALCYAEFGARVPRTGSAYLFTYVSMGELWAFLIGWNVLLEYIIGGAAVARAWSGY

LDSMFSHSIRNFTETHVGSWQVPLLGHYPDFLAAGIILLASAFVSCGARVSSWLNHTFSAISLLVILFIV

ILGFILAQPHNWSADEGGFAPFGFSGVMAGTASCFYAFVGFDVIAASSEEAQNPRRSVPLAIAISLAIAA

GAYILVSTVLTLMVPWHSLDPDSALADAFYQRGYRWAGFIVAAGSICAMNTVLLSLLFSLPRIVYAMAAD

GLFFQVFAHVHPRTQVPVAGTLAFGLLTAFLALLLDLESLVQFLSLGTLLAYTFVATSIIVLRFQKSSPP

SSPGPASPGPLTKQQSSFSDHLQLVGTVHASVPEPGELKPALRPYLGFLDGYSPGAVVTWALGVMLASAI

TIGCVLVFGNSTLHLPHWGYILLLLLTSVMFLLSLLVLGAHQQQYREDLFQIPMVPLIPALSIVLNICLM

LKLSYLTWVRFSIWLLMGLAVYFGYGIRHSKENQRELPGLNSTHYVVFPRGSLEETVQAMQPPSQAPAQD

PGHME
